# The HSP90-MYC-CDK9 network drives therapeutic resistance in mantle cell lymphoma

**DOI:** 10.1186/s40164-024-00484-9

**Published:** 2024-02-07

**Authors:** Fangfang Yan, Vivian Jiang, Alexa Jordan, Yuxuan Che, Yang Liu, Qingsong Cai, Yu Xue, Yijing Li, Joseph McIntosh, Zhihong Chen, Jovanny Vargas, Lei Nie, Yixin Yao, Heng-Huan Lee, Wei Wang, JohnNelson R. Bigcal, Maria Badillo, Jitendra Meena, Christopher Flowers, Jia Zhou, Zhongming Zhao, Lukas M. Simon, Michael Wang

**Affiliations:** 1 Center for Precision Health, School of Biomedical Informatics, The University of Texas Health Science Center at Houston, Houston, TX 77030 USA; 2 Department of Lymphoma and Myeloma, The University of Texas MD Anderson Cancer Center, Houston, TX USA; 3 Department of Pharmacology and Toxicology, University of Texas Medical Branch, Galveston, TX 77555 USA; 4 Verna and Marrs McLean Department of Biochemistry and Molecular Biology, Baylor College of Medicine, Houston, TX 77030 USA; 5 MD Anderson Cancer Center UTHealth Graduate School of Biomedical Sciences, Houston, TX 77030 USA; 6 Therapeutic Innovation Center, Baylor College of Medicine, Houston, TX 77030 USA; 7 Department of Stem Cell Transplantation and Cellular Therapy, The University of Texas MD Anderson Cancer Center, Houston, TX USA

**Keywords:** Mantle cell lymphoma, Resistance, CAR-T therapy, Single-cell RNA sequencing

## Abstract

Brexucabtagene autoleucel CAR-T therapy is highly efficacious in overcoming resistance to Bruton’s tyrosine kinase inhibitors (BTKi) in mantle cell lymphoma. However, many patients relapse post CAR-T therapy with dismal outcomes. To dissect the underlying mechanisms of sequential resistance to BTKi and CAR-T therapy, we performed single-cell RNA sequencing analysis for 66 samples from 25 patients treated with BTKi and/or CAR-T therapy and conducted in-depth bioinformatics™ analysis. Our analysis revealed that MYC activity progressively increased with sequential resistance. *HSP90AB1* (Heat shock protein 90 alpha family class B member 1), a MYC target, was identified as early driver of CAR-T resistance. *CDK9* (Cyclin-dependent kinase 9), another MYC target, was significantly upregulated in Dual-R samples. Both *HSP90AB1* and *CDK9* expression were correlated with MYC activity levels. Pharmaceutical co-targeting of HSP90 and CDK9 synergistically diminished MYC activity, leading to potent anti-MCL activity. Collectively, our study revealed that HSP90-MYC-CDK9 network is the primary driving force of therapeutic resistance.

**Supplementary Information:**

The online version contains supplementary material available at 10.1186/s40164-024-00484-9.

## Background

Mantle cell lymphoma (MCL) is an aggressive subtype of non-Hodgkin B-cell lymphoma [1]. FDA-approved Bruton’s tyrosine kinase inhibitors (BTKi, e.g., ibrutinib, acalabrutinib, and zanubrutinib) [2–4] and the CD19-targeting chimeric antigen receptor (CAR) T-cell therapy brexucabtagene autoleucel [5] represent major therapeutic milestones that have transformed MCL treatment. However, relapse frequently occurs with poor patient survival, especially for the patients with sequential resistance to BTKi and CAR-T [6, 7].

The mechanisms of ibrutinib resistance have been studied in chronic lymphocytic leukemia (CLL) [8] and MCL [9, 10], which employ distinct mechanisms to develop ibrutinib resistance. Genetic alterations occur frequently in ibrutinib-relapsed CLL patients [8], but rarely in ibrutinib-relapsed MCL patients. Instead, transcriptomic reprogramming towards OXPHOS and MYC targets appears to act as the major non-genetic driving force for ibrutinib resistance in MCL [11]. Our single-cell RNA sequencing (scRNA-seq) of MCL patient samples confirmed this and further revealed resistance-associated transcriptional heterogeneity and evolution [12]. However, the precise mechanisms underlying BTKi resistance, as well as resistance to CAR-T have not been fully understood. Given that cases with BTKi-CAR-T sequential failure have worse survival after CAR-T relapse [7], it is essential to understand the mechanisms of sequential resistance to BTKi and CAR-T therapies (BTKi-CAR-T sequential resistance) and develop alternative therapies.

Therefore, we applied single-cell RNA sequencing (scRNA-seq) to primary samples from MCL patients who developed BTKi-CAR-T sequential resistance to understand the transcriptomic evolution driving sequential resistance. We integrated two cohorts for this study: a BTKi cohort (n = 10) and a CAR-T cohort (n = 15); all patients in the CAR-T cohort had prior failure to BTKi therapy. Together with additional healthy controls, the samples collected from patients treated with these two therapies were investigated to understand the transcriptomic evolution driving BTKi-CAR-T sequential resistance at the cellular and molecular levels. Our analysis revealed outcome-associated tumor-intrinsic gene signatures, cancer hallmarks, and early-stage drivers that together indicated that the HSP90-MYC-CDK9 network drives tumor evolution and sequential resistance. Targeting this network by simultaneous inhibition of HSP90 and CDK9 showed synergistic effects in downregulation of MYC activity, thus representing a promising therapy in MCL.

## Methods

### Patient sample collection

The patient samples were collected from peripheral blood (PB), bone marrow (BM), biopsy, or apheresis (Additional file 1: Table S1). The samples were purified by Ficoll–Hypaque density gradient centrifugation and cryopreserved before processing for scRNA-seq. Most samples were collected from PB; additionally, there were 6 samples from BM (B0, D2, D4, V0, K0, and L3), 1 from lymph node (A3), and one from spleen (I2).

### Single-cell data processing and integration

The 10 × Genomics CellRanger pipeline (v6.0) [13] was used to process the raw scRNA-seq data. Reads were aligned to the UCSC human genome GRCh38 and CAR-specific sequence contigs FMC63-scFV (https://www.ncbi.nlm.nih.gov/nuccore/305690546). After generating the raw count matrix, the R package Seurat (v4.0.3) [14] was used for downstream analysis.

The samples from BTKi cohort were sequenced with single-cell 3′ gene expression kits (10 × Genomics). The samples from CAR-T cohort were subjected to simultaneous single-cell gene expression and immune profiling, thus were sequenced with single-cell 5′ kits (10 × Genomics). To enhance the statistical power and robustness of our analyses, we integrated these two cohorts to study the sequential resistance to BTKi and/or CAR-T therapy. Unsupervised dimension reduction of in silico bulk samples revealed that cell clustering was primarily dependent on the cohort, indicating a strong batch effect between two cohorts due to chemical differences in the kits (Additional file 5: Figure S1A-C). To remove the batch effect and integrate both cohorts, we applied Seurat Canonical Correlation Analysis (CCA) [15]. To enable the evaluation of batch effect correction, one sample A3 was sequenced using both 5′ and 3′-kits. The datasets from each cohort were first processed independently, and highly variable genes were identified (nfeatures = 2000). The “anchors” between two cohorts were found by the *FindIntegrationAnchors()* function in Seurat (v4.0.3). A corrected matrix was returned after removing batch effects, which became the input for the downstream dimension reduction analysis.

After CCA integration, A3 cells from the BTKi and CAR-T cohorts clustered together, demonstrating successful integration (Additional file 5: Figure S1D). Moreover, well-known cell type marker genes such as *CD8B* (CD8^+^ T cell marker) and *IL7R* (CD4^+^ T cell marker) showed concordant expression after integration, indicating that the integrated embedding removed batch effects and represented a map of bona fide cell types (Additional file 5: Figure S1E).

### Copy number variation (CNV) analysis

The *inferCNV* tool was utilized to infer CNVs for each individual cell [16]. To mitigate any bias caused by an unequal number of cells per sample, we downsampled cells to the same number of cells per sample. The copy number status in each cell was predicted by the *inferCNV* built-in six-state Hidden Markov Model (i6-HMM), including complete loss, loss of one copy, neutral, addition of two copies, and addition of more than two copies. The i6-HMM model generated a posterior probability for each chromosome region. Those with low p-values were considered as putative aberrant regions. A relatively stringent cutoff was set to include only aberrations with high confidence (BayesMaxPNormal = 0.2, default: 0.5).

### Genome instability score

The genome instability score was quantified using the CNV profiles. The matrix of inferred CNVs was subjected to principal component analysis (PCA). Next, the first 50 principal components were subjected to UMAP dimension reduction. The Euclidean distance calculated on the UMAP embedding between the normal cells and each individual cell was calculated to quantify genome instability.

### Differential expression analysis and gene set enrichment analysis

Before modeling, the raw count matrix from both cohorts was log normalized and scaled. Cell cycle scores were calculated using the *CellCycleScoring()* function in the Seurat R package (v4.0.3). The differential gene expression analysis was conducted using a linear mixed model accounting for the patient as a random effect and cell cycle scores as a fixed effect. We applied two independent regressions. For the first regression, we restricted the analysis to samples in the BTKi cohort alone. Since this cohort contained samples from all clinical outcomes, it was used to identify genes associated with each clinical outcome. The linear mixed model with random effect was expressed as follows:

1 $${\mathrm{Y}}_{{\mathrm{ij}}} = \, \beta_0 + \, \beta_{1} *{\text{ S }} + \, \beta_{2} *{\text{ G2M }} + \, \beta_{3} *{\text{ Outcome}}_{\mathrm{j}} + \, \alpha_{\mathrm{i}} + \, \varepsilon_{{\mathrm{ij}}}$$

$$\alpha_{\mathrm{i}} \ {\text{ N}}\left( {0,\sigma_{{\mathrm{patient}}}^{2} } \right)$$

$$\varepsilon_{{\mathrm{ij}}} \ {\text{ N}}\left( {0,\sigma^{2} } \right)$$

where Y_ij_ denotes the gene expression of sample j for patient i, and S and G2M are quantitative scores for the S and G2M phases. Outcome_j_ is a categorical variable representing the clinical outcome of sample j. The possible values include normal, BTKi-Fast, BTKi-Slow, BTKi-R, and Dual-R. The term β_3_ represents the fixed effect of the clinical outcome, which is of the greatest interest in our analysis. The term α_i_ denotes patient random effect and ε_ij_ denotes random error.

For the second regression, we combined data from both cohorts but restricted the analysis to Dual-R and BTKi-R samples to identify genes that were robustly altered across both cohorts. The model was expressed as follows:

2 $${\mathrm{Y}}_{{\mathrm{ij}}} = \, \beta_0 + \, \beta_{1} *{\text{ S }} + \, \beta_{2} *{\text{ G2M }} + \, \beta_{3} *{\text{ Outcome}}_{\mathrm{j}} + \, \beta_{4} *{\text{ Cohort}}_{\mathrm{j}} + \, \alpha_{\mathrm{i}} + \, \varepsilon_{{\mathrm{ij}}}$$

$$\alpha_{\mathrm{i}} \ {\text{ N}}\left( {0,\sigma_{{\mathrm{patient}}}^{2} } \right)$$

$$\varepsilon_{{\mathrm{ij}}} \ {\text{ N}}\left( {0,\sigma^{2} } \right)$$

where Outcome_j_ is a binary variable, with 0 representing BTKi-R samples and 1 representing Dual-R samples. Cohort_j_ is an indicator that equals 0 for BTKi cohort and 1 for CAR-T cohort. The p-values were adjusted using the Benjamini–Hochberg method [17]. Genes with adjusted p-values less than 0.1 were considered significant.

WebGestalt (version 0.4.4) [18] was used to run gene set enrichment analysis including multiple test correction. The cancer hallmark gene set was downloaded from the Molecular Signatures Database (MSigDB, v7.0) and contained 50 gene sets. The minimum number of genes in the pathways was set to 5 and the maximum was set to 500. The Benjamini–Hochberg method was used to adjust the p-values [17]. Those pathways with adjusted p-values less than 0.05 were considered statistically significant.

### Reanalysis of published RNA-seq data

We reanalyzed our published bulk RNA-seq dataset as described previously [11]. The R package DESeq2 (version 1.30.1) was used to perform differential expression analysis [19]. Genes with adjusted p-value less than 0.05 and absolute value of log2 fold change greater than 1 were considered as significant differentially expressed genes (DEGs). To systematically evaluate the consistency between scRNA-seq DEGs and the bulk RNA-seq DEGs, we conducted Fisher’s exact test using *fisher.test()* function in the R package stats (version 3.6.2).

### Trajectory analysis

Supervised embedding was calculated using outcome-specific genes generated by formula 1. Trajectory analysis was conducted using Monocle 3 [20] with default parameters, where the root of the trajectory was a random cell picked from the normal samples. The trajectory differential expression analysis was implemented using tradeSeq (version 1.8.0) [21], which fits a generalized additive model for every lineage using the negative binomial distribution. To identify early driver genes between trajectories, we conducted the statistical test in a specified region of the trajectories. The *earlyDETest()* function was used to test for differential expression near bifurcation points and the first two knots were selected to restrict to the region near the bifurcation point. Multiple testing correction was performed using the Benjamini–Hochberg method [17]. Genes with a false discovery rate (FDR) less than 0.05 were considered significant.

### DepMap analysis

To assess the functional relationship between HSP90 and MYC, we analyzed cell viability data from the publicly available Dependency Map (DepMap) resource. The data was downloaded from the DepMap Data portal in January 2021 (https://depmap.org/portal). Pearson correlation was used to assess the association between two genes across a selected set of cell lines. For the pan-cancer analysis, Pearson correlation was calculated across all 1,054 cell lines. For the lymphoma analysis, Pearson correlation was calculated across 35 cell lines annotated as “lymphoma” for the “lineage” variable. Lymphoma cell lines were divided into three groups based on MYC dependency tertiles. Next, we compared the HSP90 dependency between the lowest and highest two MYC dependency tertiles using a t-test.

### TCGA data analysis

We downloaded the TCGA-DLBCL RNA-seq expression matrix using the TCGAbiolinks R package [22]. Pearson correlation between the expression of *MYC* and *HSP90AB1* was calculated.

### Cell viability assay, cell apoptosis assay, and Western blotting

These assays were performed as described previously [12]. In the cell viability assay, cells were seeded at 10,000 cells per well in 96-well plates and exposed to AZD4573, zelavespib, and tanespimycin for 72 h. Subsequently, cell lysis was conducted using the CellTiter-Glo Luminescent Cell Viability Assay Reagent, and luminescence was quantified employing the BioTek Synergy HTX Multi-mode microplate reader. For the apoptosis assay, Annexin V-binding was employed. MCL cells were treated separately with the vehicle, AZD4573, zelavespib, and tanespimycin, stained with Annexin-V and propidium iodide, and then subjected to flow cytometric analysis using the Novocyte Flow Cytometer to determine the percentages of Annexin-V positive cells. Data analysis was carried out using NovoExpress or FlowJo10, and each experiment was meticulously repeated at least three times to ensure the reliability of the results, consistent with the procedures outlined in the reference.

### Bulk RNA sequencing of treatment

The Z138 cells treated with CDK9 inhibitor AZD4573 alone or in combination with HSP90 inhibitors (zelavespib and tanespimycin) at the indicated concentration for 24 h were harvested and subjected to bulk RNA sequencing. The raw files were mapped to human reference genome GRCh38 using HISAT2 [23] and quantified by StringTie [24]. We then used the R package DESeq2 (version 1.30.1) to perform differential expression analysis [19].

### Statistical analysis

All statistical analyses were conducted using R software (version 4.0.3) and GraphPad Prism (version 9). Two-sided two-sample *t*-test was used to compare differences between two groups. Results were considered statistically significant for p < 0.01 (^**^); p < 0.001 (^***^); and p < 0.0001(^****^).

## Results

### MCL patients had sequential failures to BTKi and CAR-T therapy in the clinic

**Fig. 1:**
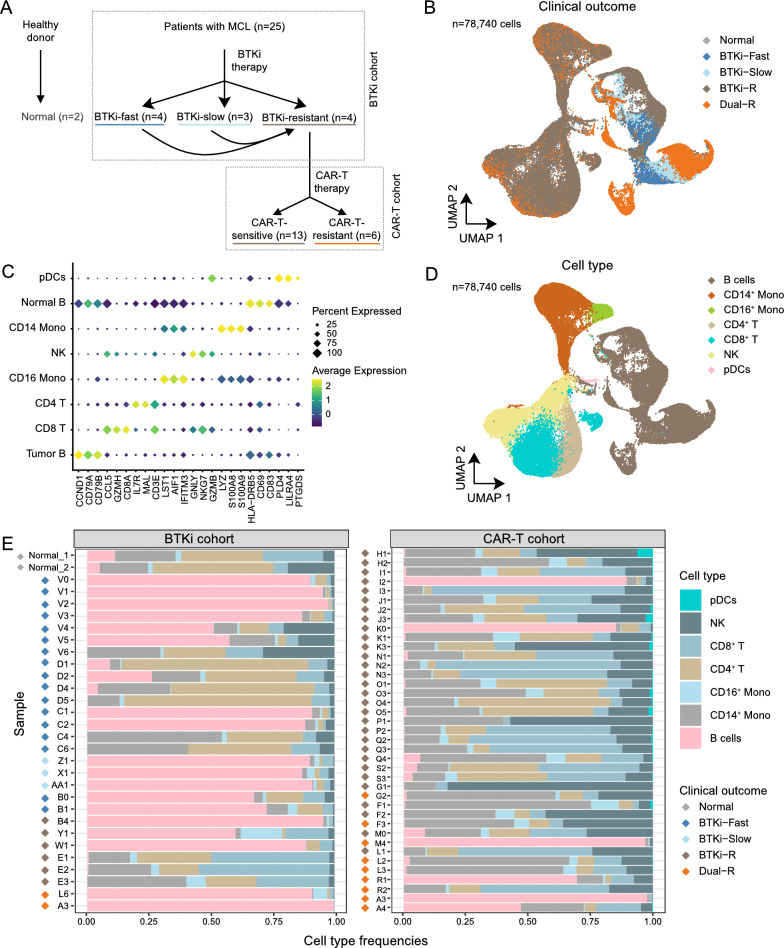
scRNA-seq reveals transcriptomic heterogeneity in MCL patients with diverse clinical outcomes. **A** Experimental design summarizing patient sample information. Patient samples were categorized into five clinical outcomes according to their sensitivity to BTKi or CAR-T therapy. The number of patients (n) were denoted in the plot. **B** UMAP visualization represents cells colored by cell type. **C** Dot plot illustrates marker gene expression across cell types. Colors indicate low (purple) to high (yellow) expression. The circle size is proportional to the percentage of cells in which the gene was expressed. **D** UMAP visualization represents cells colored by clinical outcomes. **E** Bar plot shows cell type frequencies (x-axis) of each sample (y-axis) in the BTKi (left) or CAR-T (right) cohorts. Dot in front of each sample indicate clinical outcomes

To explore the underlying mechanisms of sequential resistance to BTKi and CAR-T therapy in patients with MCL, we collected two patient cohorts in this study, designated BTKi and CAR-T (Fig. 1A). In total, we profiled 66 patient samples using scRNA-seq. Two peripheral blood mononuclear cell (PBMC) samples from healthy donors were included as normal controls. To the best of our knowledge, this dataset represents the most extensive collection of scRNA-seq data from MCL patients to date. The BTKi cohort contained 28 samples from 12 patients, designated AA, B-E, and V-Z (Additional file 1: Table S1). The CAR-T cohort contained 39 samples from 15 patients designated A and F-S. Notably, one sample, A3, was shared between both cohorts and underwent sequencing with two different kits to aid in the subsequent assessment of batch effects. Based on the clinical response and relapse stages, all samples were grouped into five clinical outcomes (Fig. 1A): (1) Normal (n = 2), (2) BTKi-Fast (collected from fast responders to BTKi therapies within 3 months post treatment; 4 patients [B (B0 and B1), C, D, and V], (3) BTKi-Slow (collected from slow responders to BTKi therapies beyond 3 months post treatment; 3 patients [X, Z, and AA]), (4) BTKi-R (collected at relapse or refractory stage from patients with failure to BTKi; 17 patients [B (B4), E, F (F1-F2), G (G1), H, I, J, K, L (L3), M (M0), N, O, P, Q, S, W, and Y]), and (5) Dual-R (collected at relapse or refractory stage from patients with sequential failure to BTKi and CAR-T therapies; 6 patients [A, F (F3), G (G2), L (L4-L5), M (M4), and R]). Part of these data have been published in a different context [12, 25]. The clinicopathological information and demographic characteristics are summarized in Additional file 2: Table S2.

### scRNA-seq captures cellular and transcriptomic heterogeneity in MCL patients

We integrated scRNA-seq data from two patient cohorts using Seurat Canonical Correlation Analysis [15] (see Methods, Additional file 5: Figure S1). In total, 78,740 single cell transcriptomes passed quality filtering (Fig. 1B). Based on canonical marker expression, seven major cell types were identified: B cells (286 normal cells, 0.36%; 33,027 tumor cells, 41.94%) and six immune cell types forming the tumor microenvironment (TME): CD4^+^ T cells (9,745 cells, 12.38%), CD8^+^ T cells (10,538 cells, 13.38%), NK cells (10,630 cells, 13.50%), CD14^+^ monocytes (12,565 cells, 15.96%), CD16^+^ monocytes (1,811 cells, 2.30%), and plasmacytoid dendritic cells (pDCs,138 cells, 0.17%) (Fig. 1C–E).

The immune cells in the TME (e.g. CD4^+^ T cells, CD8^+^ T cells, NK cells, CD14^+^ monocytes, CD16^+^ monocytes and pDCs) were clustered according to cell types (Additional file 5: Figure S2A-B, left two panels). In contrast, normal and tumor B cells were grouped into multiple distinct sub-clusters (Fig. 1D, Additional file 5: Figure S2A-B right panels), indicating high transcriptomic heterogeneity across patients even within the same clinical outcome (Additional file 5: Figure S2C). Therefore, it is important to dissect the tumor-intrinsic transcriptomic changes and molecular determinants that are responsible for the development of BTKi-CAR-T sequential resistance while accounting for the inter-patient heterogeneity.

### Increased copy number variations and proliferation reflect progression of therapeutic resistance

**Fig. 2:**
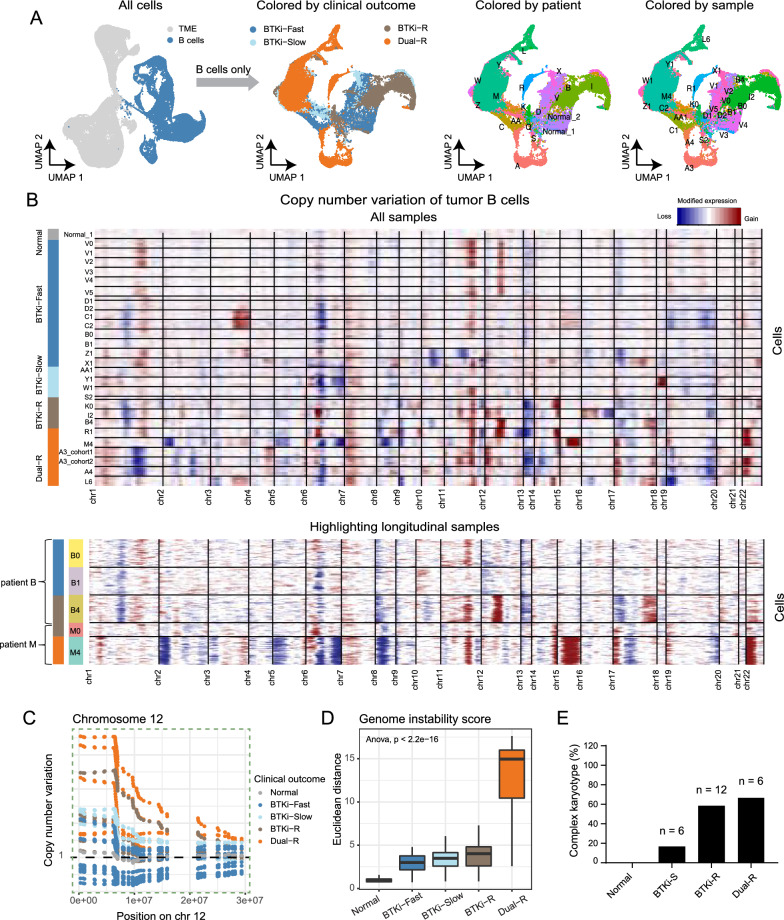
Tumor B-cell copy number variation (CNV) promotes the evolution of therapeutic resistance. **A** Left: UMAP visualization of all cells with B cells highlighted in blue. Right: UMAP visualizations of B cells colored by clinical outcomes, patient, and sample. **B** Heatmap displays cellular CNV profiles (row) of each cell across chromosomes (columns) for all samples (top) and restricted to longitudinal samples (bottom). Colors reflect copy number gains (red) and losses (blue). Sample names and clinical outcomes are annotated on the left. Samples are ordered by aggressiveness from the top (normal) to the bottom (Dual-R). **C** Plot shows the inferred copy number estimates (y-axis) for samples across chromosome 12 (x-axis). Horizontal dashed line represents expected normal copy number. **D** Boxplot shows the Euclidean distance (y-axis) derived from the CNV profile-based low dimensional space across different clinical outcomes (x-axis). **E** Percentages of complex karyotype in each clinical outcome group

To understand the transcriptomic heterogeneity in tumor cells, we first isolated cells with same cell types (e.g. B cells and CD8^+^ T cells) and investigated their transcriptomic changes at genomic level by scRNA-seq-inferred copy number variation (CNV) profiling. We applied *inferCNV* [16] to identify CNVs events using cells from healthy donors as reference (see Methods, Fig. 2A). As expected, no apparent CNVs were identified for non-tumor CD8^+^ T cells across all patient samples (Additional file 5: Figure S3A-B). In contrast, MCL tumor cells from patients showed much higher levels of CNVs compared to normal B cells from healthy donors, and the CNV profiles were distinct from each other across patients, even within the same outcome groups (Fig. 2B, top panel). *CCND1* chromosomal translocation t(11:14) is a hallmark event of MCL. Consistent with this, chromosome 11q gain was identified in the majority of tumor B cells, which is consistent with staining results (Additional file 2: Table S2). More CNVs were found in samples with resistance to BTKi and/or CAR-T compared to those sensitive to BTKi (Fig. 2B, C). For example, K0 and I2 in the BTKi-R group showed chromosome 14q gain, while B4 showed chromosome 12p and 17q gains (Fig. 2B, bottom panel); these CNVs were reported in our previous analysis and validated by whole-genome sequencing and patient-derived xenograft (PDX) models [12]. For the Dual-R group, sample R1 (CAR-T-refractory) exhibited gains in chromosomes 6p, 11q, 12p, 14q, and 22p, while M4 (CAR-T-relapsed) had losses in chromosomes 6q and 8p but gains in chromosomes 11q, 15q, 17p, and 22p. Of note, chromosome 17p and 22p gains were detected exclusively in Dual-R samples (5/6 and 4/6, respectively) (Fig. 2B, bottom panel).

To quantify the extent of CNVs for tumor cells in each sample, we calculated genome instability scores. As expected, sample A3, which was profiled in both BTKi and CAR-T cohorts for quality control purpose, presented comparable genome instability scores, demonstrating the robustness of this metric (Additional file 5: Figure S4). We observed a significant positive association between the genome instability score of each outcome group and tumor aggressiveness (ANOVA test, p < 2 × 10^–16^), with Dual-R samples having the highest scores, followed sequentially by BTKi-R, BTKi-Slow, and BTKi-Fast (Fig. 2D, and Additional file 5: Figure S4).

Complex karyotype has been identified as an important predictor of poor outcomes in patients with MCL [26]. Therefore, we assessed clinical karyotyping information available for a subset of the patients. Indeed, we found that four of six Dual-R (66.7%) and seven of twelve BTKi-R (58.3%) patients showed complex karyotypes (Fig. 2E). In contrast, only one patient (Patient V, BTKi-fast) of six BTKi-sensitive patients had a complex karyotype (16.7%). Together, these data validated our scRNA-seq-derived findings and demonstrated that accumulation of large-scale CNVs is associated with MCL tumor progression and therapeutic resistance.

**Fig. 3:**
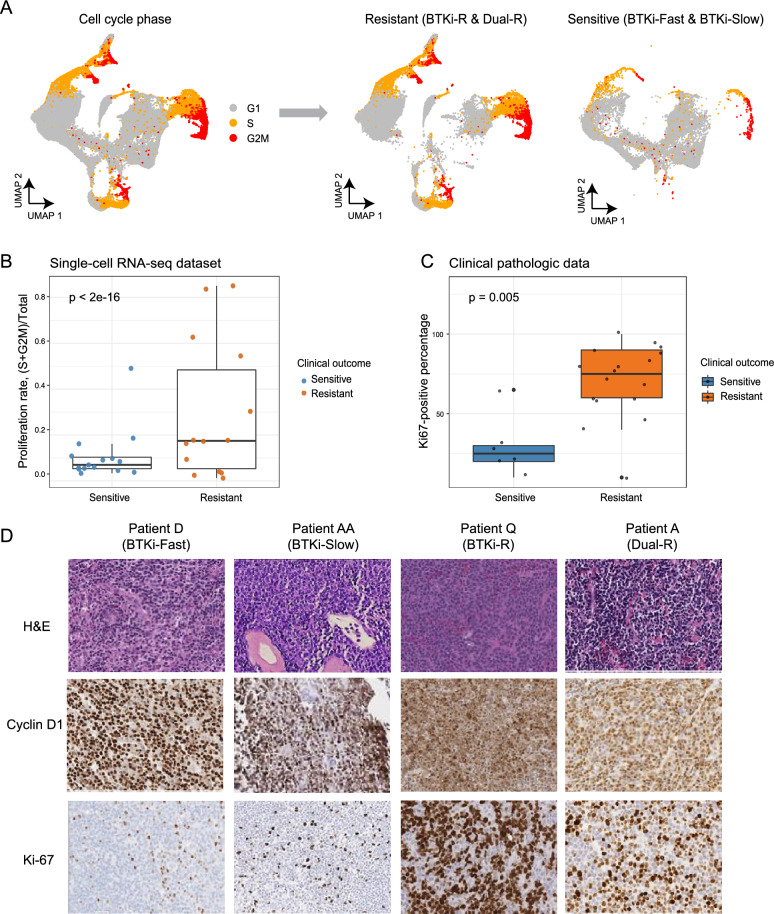
Resistant tumor cells acquire elevated proliferation rates. **A** Left: UMAP visualization of B cells colored by inferred cell cycle stages (G1, S, G2/M). Right: UMAP visualizations of B cells divided by clinical outcome: sensitive (BTKi-Fast and BTKi-Slow) and resistant (BTKi-R and Dual-R). Each dot represents one cell. Gray, orange, and red represent G1, S, and G2M cell cycle stages, respectively. **B** Boxplot shows inferred proliferation rates (y-axis) across clinical outcomes (x-axis) in single-cell RNA-seq dataset. Each dot represents one sample and is colored by clinical outcome. P-value was calculated using a generalized binomial model. **C** Boxplot shows proliferation rates as indicated by Ki-67-positive immunohistochemical staining across clinical outcomes from clinical pathologic data. Each dot represents one patient and is colored by clinical outcome. **D** Representative bone marrow images stained with hematoxylin and eosin (upper panels) or immunohistochemically stained for cyclin D1 (middle panels) or Ki-67 (bottom panels) on samples from representative patients D (BTKi-Fast), AA (BTKi-Slow), Q (BTKi-R), and A (Dual-R)

Consistent with this, we observed that the proportion of proliferating cells was highly associated with therapeutic resistance (BTKi-R or Dual-R) (Fig. 3A). Most tumor B cells from resistant samples were in S or G2/M phase, while most in the sensitive samples were in G1 phase (Fig. 3B). In patients with available pathological data from Ki-67-stained tumor biopsies, the percentages of Ki-67 positive cells were higher in resistant compared to sensitive patients (Fig. 3C, p < 2.2e-16). As representative examples, patients D (BTKi-Fast), X (BTKi-Slow), Q (BTKi-R), and A (Dual-R) all showed positive cyclin D1 staining; however, only resistant patients Q and A showed high fractions of Ki-67 staining (Fig. 3D, p = 0.005). Together, these data demonstrate that the tumor B cells in the BTKi-R or Dual-R groups acquired large-scale CNV and elevated proliferation rates that promoted disease progression.

### BTKi-CAR-T sequential resistance is reflected by specific gene expression fingerprints

**Fig. 4:**
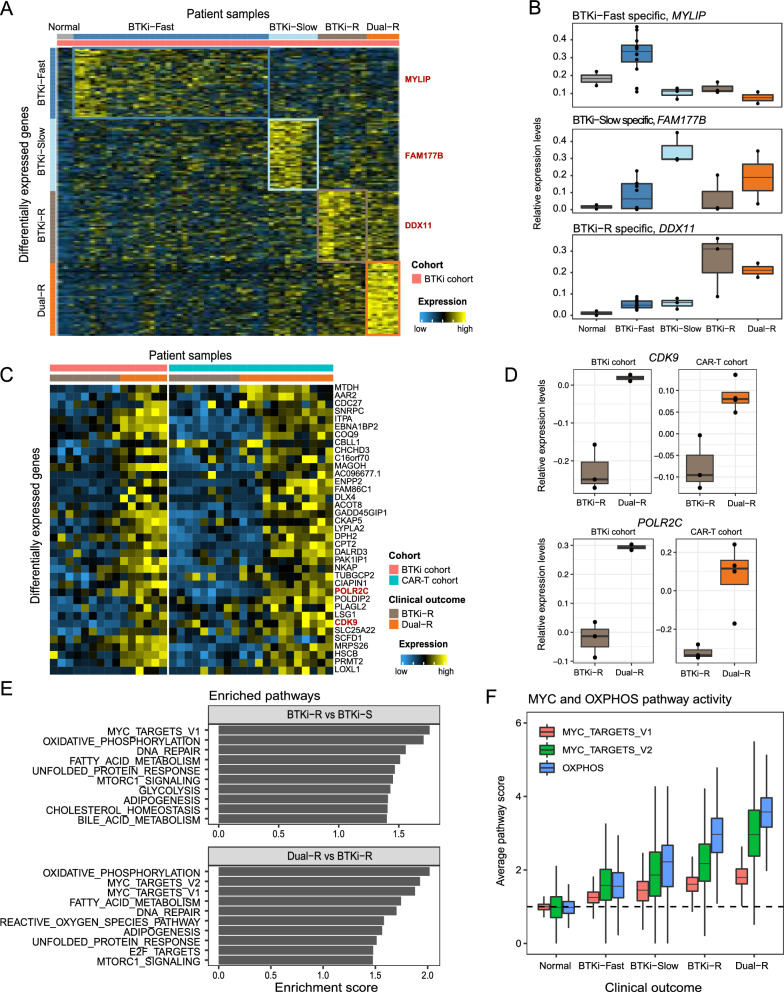
Sequential resistance to BTKi and CAR-T therapies is reflected by specific gene expression fingerprints. **A** Heatmap shows the expression profile of outcome-specific genes (rows) across samples. Columns represent averaged expression profile of random 10 cells for each sample. Bars on the top denote clinical outcomes (five groups). Bars on the left highlight the outcome specific genes (four groups). **B** Boxplots show the expression of three outcome-specific gene expression across samples for representative genes *MYLIP*, *FAM177B,* and *DDX11*. Each dot represents averaged expression profile of random 10 cells for each sample. **C** Heatmap shows the expression profile of genes (rows) with significant changes between the Dual-R and BTKi-R samples across both cohorts. Columns represent averaged expression profile of random 10 cells for each sample. Representative genes (*CDK9* and *POLR2C*) are highlighted in red. **D** Boxplots show differential expression of *CDK9* and *POLR2C* in Dual-R and BTKi-R samples of both cohorts. Each dot represents averaged expression profile of random 10 cells for each sample. **E** Bar plots summarize the enriched pathways in different contrasts. Top: BTKi-R vs BTKi-sensitive (BTKi-Fast/Slow). Bottom: Dual-R vs BTKi-R. **F** Boxplots show average pathway scores (y-axis) of MYC_TARGETS_v1, MYC_TARGETS_v2, and OXPHOS gene sets across clinical outcomes (x-axis). There is a progressive enrichment of MYC targets and the OXPHOS pathway across the clinical outcomes

We next performed differential expression analysis to detect outcome-specific gene expression signatures. Due to high tumor heterogeneity across patients, we applied a mixed model with random effect accounting for patient heterogeneity, which outperformed alternative differential expression analysis approaches (Additional file 5: Figure S5). Because therapeutic-resistant cells are highly proliferating (Fig. 3), cell proliferation rate was included in the model as covariate to account for this effect. We first examined the BTKi cohort alone and identified distinct gene expression signatures associated with each BTKi-CAR-T clinical outcome (Fig. 4A, Additional file 3: Table S3). For example, expression of *MYLIP, FAM177B,* and *DDX11* was upregulated in BTKi-Fast, BTKi-Slow, and BTKi-R samples, respectively (Fig. 4B). Despite high levels of patient heterogeneity our analysis identified several genes that were significantly upregulated across most patients for each outcome even after correcting for multiple testing. Visualization of the distribution of p-values derived from our regression models revealed strong enrichment of low p-values, demonstrating the presence of statistical signal that goes beyond spurious associations (Additional file 5: Figure S6).

To validate our outcome-specific gene signatures, we reanalyzed publicly available bulk RNA-seq data from an independent cohort containing 6 ibrutinib-resistant (BTKi-R) and 15 ibrutinib-sensitive (BTKi-S) samples [11]. The bulk RNA-seq data was obtained from MCL primary patient samples while our regression models were restricted to tumor B cells. Despite these differences in cellular composition, we observed a significant correlation of fold changes between our scRNA-seq and bulk RNA-seq data (Additional file 5: Figure S7, Pearson correlation Rho: 0.15, p = 9.20 × 10^–30^). For example, *GSAP* and *PRMT1* were down- and up-regulated, respectively, in BTKi-R compared to BTKi-S samples in both the scRNA-seq and bulk RNA-seq data (Additional file 5: Figure S7).

Next, we combined the BTKi and CAR-T cohorts to examine genes with robust differential expression between BTKi-R and Dual-R samples in both BTKi and CAR-T cohorts. We identified 37 genes that were robustly upregulated in Dual-R samples compared to BTKi-R samples across both cohorts passing multiple testing correction (Fig. 4C, linear mixed model, adjusted p-value < 0.1). Among them, genes involved in transcription machinery (e.g., *POLR2C*), transcription regulators (e.g., *CDK9* and *PRMT2*), and transcription factors (e.g., *CHCHD3*, *DLX4*, and *PLAGL2*) were upregulated in Dual-R compared to BTKi-R samples (linear mixed model, adjusted p-value < 0.1), suggesting a reprogramming towards increased transcription associated with Dual-R (Fig. 4D). Importantly, all of the above genes are targets of the master regulator MYC [27]. For example, CDK9 is critical for the continuous expression of genes producing short-lived mRNAs or proteins, such as MYC and MCL-1 that promote cancer cell survival [28].

We then performed gene set enrichment analysis (GSEA) to assess the functions of outcome-associated gene signatures. Compared to BTKi responders (BTKi-Fast and BTKi-Slow), the BTKi-R patients were enriched for MYC_TARGETS_v1, OXPHOS, DNA repair, and fatty acid metabolism (FDRs < 0.05), which were further enriched in Dual-R samples compared to BTKi-R samples (Fig. 4E, Additional file 4: Table S4). Our analysis identified progressive enrichment of MYC targets and OXPHOS pathway across clinical outcomes, which were associated with BTKi-CAR-T sequential resistance (Fig. 4F, Additional file 4: Table S4). Altogether, these data highlighted the role of MYC targets in contributing to sequential resistance, which may represent a novel therapeutic entry-point for overcoming resistance to BTKi and/or CAR-T therapy.

### Pseudotemporal expression analysis reveals early drivers of therapeutic resistance

**Fig. 5:**
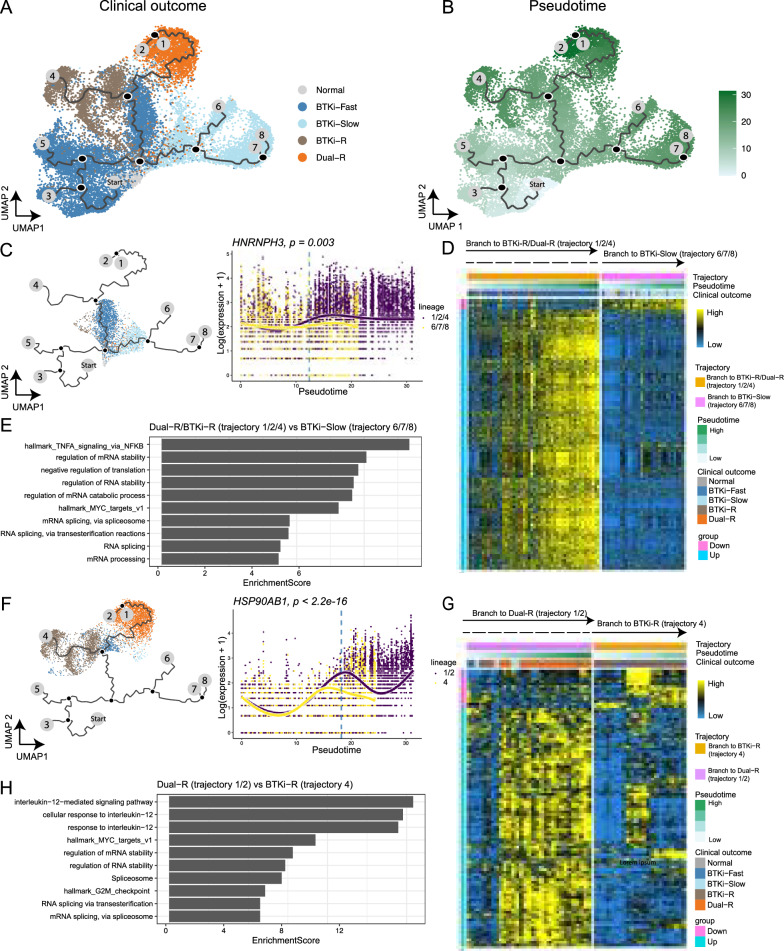
Pseudotemporal analysis reveals early-stage drivers acting on therapeutic resistance. **A** UMAP visualization illustrates inferred trajectories. Starting and end points are labeled with gray circles. Branch points are shown in black circles. Each dot represents one cell and is colored according to clinical outcome. **B** UMAP visualization colored by inferred pseudotime. (**C**) Left: UMAP visualization of cells used for comparison of BTKi-R/Dual-R (1/2/4) and BTKi-Slow (6/7/8) trajectories. Right: Scatter plot with fitted smooth curves shows the expression of top hit *HNRNPH3* across pseudotime in the BTKi-R/Dual-R (1/2/4, purple) and BTKi-Slow (6/7/8, yellow) trajectories. A vertical dashed blue line marks the pseudotime at the branch point. **D** Heatmap shows the expression pattern of differentially expressed genes (rows) at the bifurcation point between the BTKi-R/Dual-R (1/2/4) and BTKi-Slow (6/7/8) trajectories (columns). Columns are ordered by trajectory with increasing pseudotime. Blue and yellow colors represent low and high expression, respectively. Bars on top illustrate clinical outcome, pseudotime, and inferred trajectories. **E** Gene set enrichment analysis summarizes the top enriched pathways. x-axis: normalized enrichment score. (**F–H**) Similar visualization as in **C–E**, focusing on the comparison of the Dual-R (1/2) and BTKi-R (4) trajectories

To understand the early-stage transcriptomic changes leading to the emergence of therapeutic resistance, we performed pseudotemporal trajectory analysis [20, 29]. The analysis revealed eight trajectories with distinct patient outcomes at the termini (Fig. 5A). The major trajectory stemmed from normal to BTKi-Fast samples, then branched into BTKi-Slow and BTKi-R/Dual-R samples, with the latter further branching into BTKi-R and Dual-R samples. As expected, Dual-R samples had the largest pseudotime values, indicating that Dual-R cells showed the largest transcriptomic differences from normal B cells (Fig. 5B).

To find early drivers of BTKi-CAR-T sequential resistance, we performed pseudotemporal gene expression analysis. Distinct from the gene expression analysis (Fig. 4), pseudotemporal gene expression analysis identified genes altered near trajectory branch points, which reveal early drivers of distinct trajectories [21]. We first focused on cells near the bifurcation point separating BTKi-Slow (trajectory 6/7/8) from BTKi-R (trajectory 1/2/4) samples (Fig. 5C). In total, 335 genes were upregulated and 20 genes were downregulated in BTKi-R compared to BTKi-Slow cells (adjusted p < 1 × 10^–6^) (Fig. 5D, Additional file 4: Table S4). Among the upregulated genes, we discovered several heterogeneous nuclear ribonucleoprotein (hnRNP) genes: *HNRNPH3*, *HNRNPDL*, *HNRNPR*, *HNRNPC*, and *HNRNPA2B1* (Fig. 5C). Interestingly, all these genes are MYC targets that regulate RNA metabolism, including alternative splicing, mRNA stabilization, transcription, and translation pathways [30]. GSEA revealed strong enrichment of TNFα signaling via NF-κB, MYC_TARGETS_v1, multiple RNA metabolism processes including RNA splicing, and regulation of mRNA stability (Fig. 5E).

Next, we focused on early drivers of CAR-T resistance. A comparison of cells near the bifurcation point separating Dual-R (trajectory 1/2) and BTKi-R (trajectory 4) samples yielded 354 differentially expressed genes (adjusted p < 1 × 10^–6^, Fig. 5G). The majority of these genes were upregulated in Dual-R versus BTKi-R samples, including heat shock protein genes *HSP90AB1* (Fig. 5F) and *HSP90AA1* (not shown)*,* which are involved in protein folding and have been shown to promote cancer cell proliferation and migration [17, 18]. GSEA also revealed upregulation of MYC_TARGETS_v1, G2M_checkpoint, and regulation of mRNA stability (FDRs < 0.05, Fig. 5H) as early signaling pathways driving the development of CAR-T resistance.

### Coordination between HSP90, MYC, and CDK9 drives therapeutic resistance

**Fig. 6:**
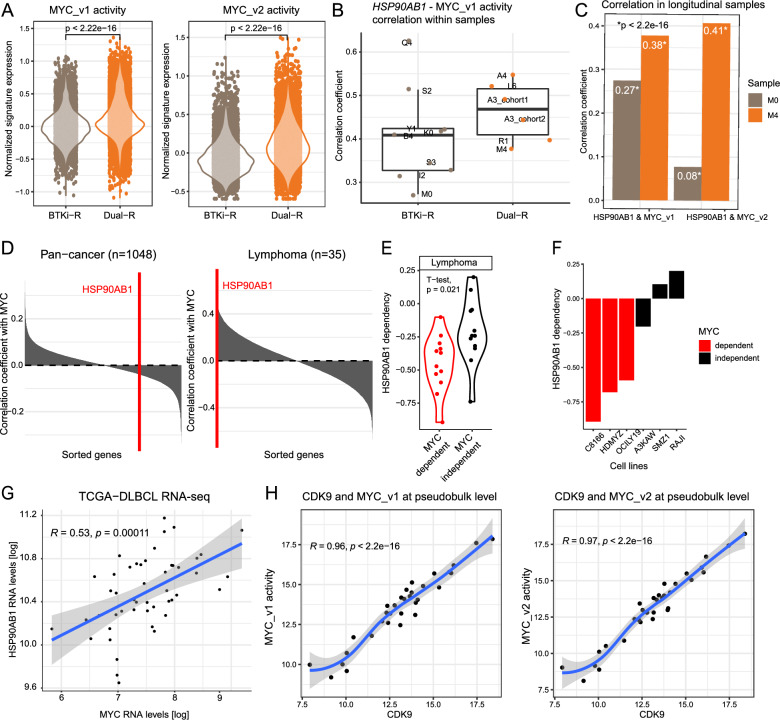
Coordination of HSP90, MYC, and CDK9 drives therapeutic resistance. **A** Violin plots show inferred cellular pathway activity of MYC_TARGETS_v1 and MYC_TARGETS_v2 across the BTKi-R and Dual-R groups. *HSP90AB1* is a part of the MYC_TARGETS_v1 gene set. To avoid bias, we removed it from the MYC_TARGETS_v1 gene set. **B** Boxplots show intra-sample correlation coefficients between *HSP90AB1* expression and MYC_TARGETS_v1 activity in the BTKi-R and Dual-R groups. Each dot represents the correlation between *HSP90AB1* expression and MYC_TARGETS_v1 activity across the individual cells within a single sample. **C** Barplots show increased correlation between *HSP90AB1* and MYC activities in longitudinal samples. **D** Plots show the correlation between *HSP90AB1* and *MYC* dependencies (y-axis) for all genes in DepMap (y-axis) across all cancer cell lines (top) and restricted to lymphoma cell lines (bottom). Red vertical line marks the position of *HSP90AB1* in genome-wide ranking of genes based on correlation with *MYC*. **E** Violin plots show increased *HSP90AB1* dependency (y-axis) across lymphoma cell lines divided into *MYC*-dependent and -independent groups (x-axis). Low values indicate greater dependency. **F** Barplot shows HSP90AB1 dependency (y-axis) across select lymphoma cell lines (x-axis). Colors indicate MYC dependency. **G** Scatter plot shows the correlation of *MYC* and *HSP90AB1* RNA-seq expression across tumors in the TCGA DLBCL cohort. **H** Scatter plot shows the correlation of *CDK9* expression and MYC activities at pseudobulk level

The above differential gene expression analysis suggested a progressive increase of expression of MYC targets with sequential resistance (Fig. 4F). In addition, our trajectory analysis revealed two HSP90 genes (*HSP90AB1* and *HSP90AA1*) that are both MYC targets as the top early-stage driver genes linked to the development of CAR-T resistance beyond BTKi resistance (Fig. 5F). Considering the known function of HSP90 in mediating MYC stability [31] and work that postulated HSP90 as a drug target in MYC-driven B-cell lymphoma [32], we hypothesized that a coordinated regulation between HSP90 and MYC (and its targets) drives the development of CAR-T resistance. To test this hypothesis, we inferred MYC activity by quantifying the aggregate expression levels of MYC targets and found they were significantly higher in Dual-R compared to BTKi-R cells (Fig. 6A).

Next, we found that the correlation between *HSP90AB1* expression and MYC activity levels across individual cells was higher in the Dual-R samples (p < 2.2 × 10^–16^) (Fig. 6B). Our longitudinal sampling allowed us to assess the changes in the correlation before and after relapse within the same patient. For example, we observed a strong increase in the correlation between HSP90AB1 expression with MYC activities in patient M, who transitioned from CAR-T sensitive (M0) to resistant (M4) (Fig. 6C). These results linked increased coordination between HSP90 expression and MYC activity to BTKi-CAR-T sequential resistance.

To assess the functional relationship between HSP90 and MYC, we analyzed CRISPR-Cas9-based cell viability screen data from the publicly available Dependency Map (DepMap, https://depmap.org/portal). We downloaded cell viability measurements following genome-wide CRISPR-Cas9 loss-of-function screens across 17,386 genes and 1,054 cell lines of various cancer types. Those genes whose perturbation caused a decrease in cell viability received negative dependency scores, which may suggest their roles in potential cancer-specific dependencies.

To discover potential relationships [33], we calculated the correlation between MYC dependency and all other genes on a pan-cancer level. We observed no significant association between the dependencies of MYC and *HSP90AB1* (Fig. 6D, left panel). However, when restricting the analysis to lymphoma cell lines only, we observed a significant correlation between MYC and *HSP90AB1* dependencies that was among the strongest MYC correlations on a transcriptome-wide level (Pearson correlation, *Rho* = 0.44, p = 0.0091, Fig. 6D, right panel, and Additional file 5: Figure S8). Indeed, when separating lymphomas into MYC-dependent and -independent cell lines, we observed that HSP90AB1 dependency scores were significantly lower in MYC-dependent compared to MYC-independent cell lines, indicating a potential relationship between HSP90AB1 and MYC (*t*-test, p = 0.021, Fig. 6E). For example, cell lines C8166, HDMYZ and OCIULY19, which are among the most MYC dependent lymphoma lines showed increased dependency compared to A3KAW, SMZ1 and RAJI (Fig. 6F). Consistent with this, HSP90AB1-MYC correlation was also confirmed using DLBCL patient samples (n = 47) from TCGA database (p = 0.00011, Fig. 6G). Likewise, we also observed high correlation between *CDK9* expression and MYC activity levels across samples at pseudobulk levels in MCL cells (Fig. 6H).

### Targeting the HSP90-MYC-CDK9 signaling network to overcome therapeutic resistance

To validate our hypothesis, we first assessed HSP90 and CDK9 protein expression in MCL primary patient cells and cell lines using western blotting. Although these proteins can be detected in most primary patient cells and cell lines, their expression was much higher in Dual-R compared to CAR-T-naïve samples (Additional file 5: Figure S9A). AZD4573, a CDK9 inhibitor currently under clinical investigation (NCT03263637), was highly potent against all MCL cell lines tested, with an IC_50_ value of only 4.0–16.6 nM (Additional file 5: Figure S9B). AZD4573 treatment markedly suppressed expression of short-lived proteins, especially MYC and MCL-1 and MCL cell viability in dose- and time-dependent manner while apoptosis markers PARP cleavage and caspase 3 cleavage were greatly induced (Additional file 5: Figure S9C-D). Similar to CDK9 inhibition, both HSP90 inhibitors zelavespib and tanespimycin could effectively induce anti-MCL activity in dose-dependent manner (Additional file 5: Figure S9E). Upon HSP90 inhibition, protein expression of MYC and CDK9 was markedly reduced (Additional file 5: Figure S9F). As expected [34], HSP90 expression was upregulated upon HSP90 inhibition.

To understand the mechanisms of anti-MCL action of CDK9 inhibition and HSP90 inhibition, we performed bulk RNA sequencing for Z138 cells treated with AZD4573 (2.5 and 5 nM), zelavespib (0.2 and 0.4 µM) and tanespimycin at (0.5 and 1.0 µM) at low doses. The top signaling pathway downregulated by AZD4573 at both low doses was TNFα signaling via NF-κB (Additional file 5: Figure S10). In contrast, the top signaling pathways downregulated by HSP90 inhibitors at both low doses were E2F targets and G2M checkpoint. Unexpectedly, TNFα signaling via NF-κB was the top signaling pathways upregulated by both HSP90 inhibitors at either dose (enrichment ratio = 3.82, FDR = 1.3e-13, Additional file 5: Figure S10). This suggested that the upregulated TNFα signaling via NF-κB, as a compensatory survival signal, likely play a critical to promote MCL cell survival upon treatment HSP90 inhibition and therefore compromise the efficacy of anti-MCL activity of HSP90 inhibitors. Therefore, one would expect much stronger anti-MCL activity when both CDK9 and HSP90 are inhibited even at the low doses.

**Fig. 7:**
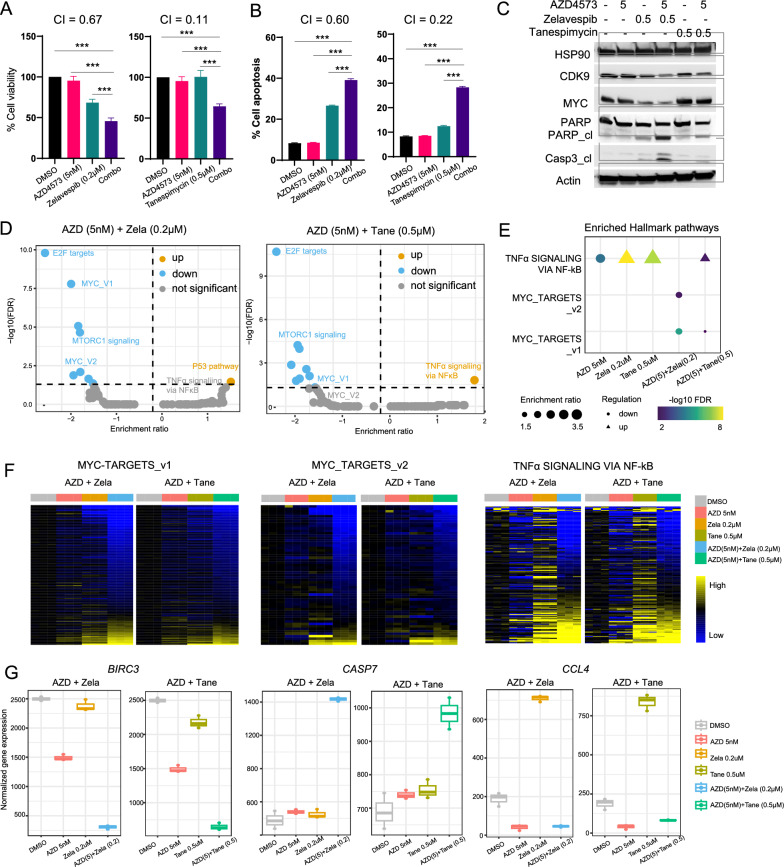
Combined treatment of CDK9 and HSP90 inhibitors shows synergistic potent anti-MCL activity. **A-B** AZD4573 in combination with zelavespib or tanespimycin synergistically suppressed cell viability (A) and induced apoptosis (B) in Z138 cells upon treatment for 72 h. CI = (I_d1_ + I_d2_)/I_(d1+d2)_. CI, combination index; I_d1_, the percentage of viability inhibition or apoptosis induction by drug #1 treatment; I_d2_, the percentage of viability inhibition or apoptosis induction by drug #2 treatment; I_(d1+d2)_, the percentage of viability inhibition or apoptosis induction by combination treatment of drug #1 and #2. The combination effect is considered synergistic if CI < 0.9. **C** Western blot shows HSP90 inhibitors zelavespib and tanespimycin in combination with CDK9 inhibitor AZD4573 induced marked reduction of MYC expression and cleavage of PARP and caspase 3. **D** Volcano plot shows the log2 fold change (x-axis) and -log10 adjusted p-value (y-axis) of enriched pathways in different treatments. Left: at 5 nM plus zelavespib at 0.2 µM. Right: AZD4573 at 5 nM plus tanespimycin at 0.5 µM. Each dot represents an enriched pathway and is colored by significance (up: yellow, down: blue, not significant: grey). **E** Dot plot shows significantly enriched hallmark pathways (y-axis) for each group (x-axis) compared to control (DMSO). Dot shape represent regulation direction (circle: down, triangle: up). **F** Heatmaps display expression of genes from relevant pathways (rows) across conditions (columns). Data was normalized to the vehicle (DMSO) condition. Blue and yellow reflect low and high expression, respectively. Dual targeting of HSP90 and CDK9 markedly suppressed MYC_TARGETS_v1 (left), MYC_TARGETS_v2 (middle), and NF-κB targets (right). **G** Boxplots show representative genes altered upon treatment with CDK9 inhibitor AZD4573 and HSP90 inhibitors, alone or in combination. ^**^, p < 0.01; ^***^, p < 0.001; ^****^, p < 0.0001

Indeed, when Z138 cells were treated with AZD4573 (5 nM) in combination with either zelavespib (0.2 µM) or tanespimycin (0.5 µM), we observed strong synergy in inhibiting cell viability (CI = 0.67 and 0.11, respectively) and in inducing cell apoptosis (CI = 0.60 and 0.22, respectively) (Fig. 7A-B). Five additional MCL cell lines were used to assess the combination effect, consistently demonstrating similar results (Additional file 5: Figure S11). The combined treatment reduced protein expression of MYC and CDK9 and induced PARP cleavage and Caspase 3 cleavage beyond each single agent (Fig. 7C). Additional bulk RNA sequencing of combined treated Z138 cells (AZD4573 at 5 nM plus zelavespib at 0.2 µM, AZD4573 at 5 nM plus tanespimycin at 0.5 µM) showed that the top signaling pathway downregulated by dual targeting of CDK9 and HSP90 is E2F targets. Interestingly, the combinations markedly altered expression of MYC targets beyond single agents (Fig. 7D-F). More interestingly, TNFα signaling via NF-κB, the top signaling pathway upregulated by both HSP90 inhibitors, are reduced by the combinations to a non-significant level (FDR > 0.05) or a less enriched level (enrichment ratio = 1.98, FDR = 0.015) (Fig. 7F). For example, *BIRC3*, a MYC target and a member of the inhibitor of apoptosis family, was strongly downregulated by the combination treatments, but this effect was weak when treated with any single agent (Fig. 7G, left panel). In contrast, expression of caspase 7 (*CASP7*), a MYC target and an apoptosis executioner, is highly induced by the combination treatments, but only weakly by single agents (Fig. 7G, middle panel). Expression of *CCL4*, a target of NF-κB signaling, was downregulated upon CDK9 inhibition and highly upregulated upon HSP90 inhibition, compared to DMSO-treated controls; however, its expression was downregulated upon dual inhibition of CDK9 and HSP90, compared to DMSO-treated controls (Fig. 7G, right panel). Together, these data suggested that combined inhibition of CDK9 and HSP90 demonstrated synergistic effects on transcriptional changes leading to anti-MCL activity.

## Discussion

In this study, we conducted scRNA-seq analysis to investigate the underlying mechanisms of sequential resistance to BTK inhibitors (BTKi) and CAR-T therapy in MCL. We identified robust gene expression signatures associated with both BTKi and CAR-T resistance. Notably, we detected a list of differentially expressed genes, including CDK9, which plays a critical role in gene transcription. Gene set enrichment analysis revealed a progressive enrichment of MYC targets, suggesting that MYC has a central role in driving sequential resistance. Our pseudotemporal trajectory analysis indicated that HSP90 genes are early-stage drivers that distinguish Dual-R and BTKi-R samples. These findings were supported by experimental data, collectively highlighting the potential importance of the HSP90-MYC-CDK9 network in driving tumor evolution and sequential resistance.

MYC emerged as a key player in our investigation. It is a proto-oncogene known to regulate a multitude of genes involved in crucial cellular functions, including cell survival, growth, proliferation, metabolism, and biogenesis, in various cancer types [35]. For instance, MYC translocations are characteristic of aggressive lymphoma subtypes like Burkitt lymphoma, diffused large B-cell lymphoma, and follicular lymphoma [36, 37]. Although MYC translocation does not occur frequently in MCL, the overexpression and upregulation of MYC targets are associated with aggressiveness and therapeutic resistance, including BTKi-resistance [11, 12] and CAR-T resistance (this study).

While MYC has traditionally been considered an "undruggable" oncogene, there has been substantial progress in developing indirect MYC-targeting strategies, both in preclinical research and clinical investigations. This development has been spurred by the recognition that MYC mRNA and protein both have very short half-lives, approximately 10 min and 20–30 min, respectively [38, 39]. Therefore, the acute and effective suppression of MYC expression and its downstream targets can be achieved by targeting the hyperactive transcription machinery in tumor cells. Inhibitors of key transcription regulators like BRD4, CDK7, and CDK9 have shown significant potential [40]. CDK9, in particular, plays a critical role as the gatekeeper of transcription productivity [28] and has direct links to the regulation of MYC. Targeting CDK9 induces acute loss of MYC expression and potent cell apoptosis in many cancer models, and it has been reported to induce vulnerability in ibrutinib-resistant MCL cells [41]. Clinical trials are underway to assess the safety and efficacy of targeting CDK9 with AZD4573 or VIP-152 in hematological malignancies (e.g., NCT04978779 and NCT03263637) and other advanced cancer types.

In this work, we also identified *HSP90AB1* and *HSP90AA1* as the top early-stage drivers of CAR-T resistance following BTKi resistance. Both genes are members of the HSP90 family and are functionally involved in protein folding and degradation. Importantly, MYC is a binding partner of HSP90 [32]. Inhibition of HSP90 suppresses MYC expression in MYC-driven Burkitt lymphoma [42] and BTKi-resistant MCL [32]. Moreover, our analysis of CRISPR-Cas9-based loss-of-function screens demonstrated potential relationship between *HSP90* and *MYC*, which is specific to lymphoma and not observable at the pan-cancer level. Consistent with this, we showed that *HSP90* expression significantly correlates with MYC activity in MCL. These data uncovered an important role of HSP90-MYC coordination in driving lymphoma.

HSP90 has long been considered as a promising anti-tumor target. Multiple inhibitors (> 18) with desirable preclinical efficacy and pharmacological properties (e.g. zelavespib, tanespimycin and AUY922) have been developed and evaluated clinically in solid cancer and even in lymphoma (e.g. NCT02572453 with AUY922 in lymphoma) [43]. However, as a single agent, none of them showed exciting clinical efficacy in treating cancer patients. These demonstrated that targeting HSP90 alone is not an ideal therapeutic strategy to treat cancer. Consistent with prior research [44], our study also reveals the upregulation of HSP90 expression upon HSP90 inhibition. This is likely attributed to the compensatory upregulation of survival signaling, such as HSF1-mediated heat shock response (HSR), triggered by HSP90 inhibition. Interestingly, CDK9 inhibition has been to be potent inhibitors of HSF1-mediated HSR. Therefore, CDK9 inhibition in combination with HSP90 inhibition will prevent protective HSF1-mediated HSR and cell survival in cancer cells. However, this is not the case in MCL cells.

Based on the data above, we propose the following model. In BTKi-R MCL cells, overexpressed HSP90 functions to ensure the folding of MYC protein, which leads to MYC protein overexpression and consequentially aberrant expression of MYC targets, including HSP90 genes and transcriptomic reprogramming. In Dual-R cells, overexpressed CDK9 further facilitates productive transcription triggered by HSP90-MYC signaling pathways, leading to extended expression of MYC and its targets, and transcriptomic reprogramming.

## Conclusion

In conclusion, our study provides novel insights into the mechanisms of BTKi-CAR-T sequential resistance in MCL. We have established that MYC and the HSP90-MYC-CDK9 network play pivotal roles in driving therapeutic resistance and transcriptomic reprogramming in MCL. While MYC has traditionally been challenging to target directly, indirect approaches like CDK9 inhibition show promise and are currently undergoing clinical evaluation. Additionally, HSP90 emerges as an early-stage driver of CAR-T resistance, and its inhibition may hold therapeutic potential. Our findings suggest a dual inhibition strategy targeting both HSP90 and CDK9 as a novel therapeutic approach to overcome sequential resistance in MCL. This strategy has the potential to improve anti-MCL activity beyond the effectiveness of single agents. Moreover, the insights gained from this study may have broader implications for addressing resistance in other MYC-driven cancer types. Overall, our research underscores the importance of understanding the complex molecular networks that underlie resistance mechanisms and the potential for innovative therapeutic strategies to combat them.

## Supplementary Information

**Additional file 1.** Sample information. The table provides information on the cohort, patient details, sample source, clinical outcomes, and days post BTKi treatment for each sample.

**Additional file 2.** Patient characteristics. The table provides information on patient characteristics, such as age, sex, Splenomegaly, ki67, p53 deletion.

**Additional file 3.** Outcome_specific_DEG. This file lists the results from the mixed model identifying genes with specific expression in each clincal outcome.

**Additional file 4.** EarlyDrivers_of_differentiation. This file contains the results for the early driver trajectory analysis.

**Additional file 5.** Additional figures. **Figure S1.** Data integration removes batch effects.** Figure S2.** Tumor B cells exhibit increased inter-patient heterogeneity compared to the tumor microenvironment. **Figure S3.** Tumor B cells carry more CNV aberrations compared to non-tumor T cells. **Figure S4.** Higher genome instability scores associate with sequential resistance to BTKi and CAR-T therapy. **Figure S5.** Mixed model detected outcome-specific gene signatures across multiple patients and samples. **Figure S6.** Histograms showing distribution of p-values derived from our mixed regression models. **Figure S7.** Outcome-specific gene signature was validated in an independent bulk RNA-seq MCL dataset. **Figure S8.** Positive Correlation between MYC and HSP90AB1 is tissue-specific and only in lymphoma cell lines. **Figure S9.** Altered pathways upon single treatment of CDK9 or HSP90 inhibitors. **Figure S10.** Enriched pathways upon single inhibitor treatment. **Figure S11.** The combination effect of CDK9 or HSP90 inhibitors were validated in five additional MCL cell lines.

## Data Availability

All R scripts supporting the findings of this paper are available on github (https://github.com/lkmklsmn/b_cell_lymphoma/). Part of the single-cell expression data used in this study has been deposited in the European Genome-Phenome Archive (EGA) database under the accession code EGAS00001005019 and we are still in process of data deposition for the rest of the sequencing data. Request for the relevant data can be made to Dr. Michael Wang at miwang@mdanderson.org. In addition to the datasets generated internally for this study, we downloaded the CRISPR-Cas9 screen dataset from DepMap (https://ndownloader.figshare.com/files/34990033). The details can be found from https://doi.org/10.1101/720243. The cancer hallmark gene sets were downloaded from MSigDB (https://www.gsea-msigdb.org/gsea/msigdb/index.jsp). All the other data are available from the corresponding authors upon request.

## Abbreviations

MCL: Mantle cell lymphoma
BTKi: Bruton’s tyrosine kinase inhibitors
CAR: Chimeric antigen receptor
HSP90AB1: Heat shock protein 90 alpha family class b member 1
CDK9: Cyclin-dependent kinase 9
CLL: Chronic lymphocytic leukemia
scRNA-seq: Single-cell RNA sequencing
PB: Peripheral blood
BM: Bone marrow
CCA: Canonical correlation analysis
PCA: Principal component analysis
MSigDB: Molecular signatures database
DEGs: Differentially expressed genes
DepMap: Dependency map
PBMC: Peripheral blood mononuclear cell
TME: Tumor microenvironment
CNV: Copy number variation
PDX: Patient-derived xenograft
GSEA: Gene set enrichment analysis
hnRNP: Heterogeneous nuclear ribonucleoprotein
HSR: Heat shock response

## References

[1] Jain P, Wang ML. Mantle cell lymphoma in 2022-A comprehensive update on molecular pathogenesis, risk stratification, clinical approach, and current and novel treatments. Am J Hematol. 2022;97(5):638–56. 35266562 10.1002/ajh.26523

[2] Wang ML, Rule S, Martin P, Goy A, Auer R, Kahl BS, et al. Targeting BTK with ibrutinib in relapsed or refractory mantle-cell lymphoma. N Engl J Med. 2013;369(6):507–16. 23782157 10.1056/NEJMoa1306220 PMC4513941

[3] Wang M, Rule S, Zinzani PL, Goy A, Casasnovas O, Smith SD, et al. Acalabrutinib in relapsed or refractory mantle cell lymphoma (ACE-LY-004): a single-arm, multicentre, phase 2 trial. Lancet. 2018;391(10121):659–67. 29241979 10.1016/S0140-6736(17)33108-2 PMC7864374

[4] Song YQ, Zhou KS, Zou DH, Zhou JF, Hu JD, Yang HY, et al. Safety and activity of the investigational Bruton tyrosine kinase inhibitor zanubrutinib (BGB-3111) in patients with mantle cell lymphoma from a phase 2 trial. Blood. 2018;132(Suppl 1):148.29866818

[5] Wang M, Munoz J, Goy A, Locke FL, Jacobson CA, Hill BT, et al. KTE-X19 CAR T-cell therapy in relapsed or refractory Mantle-Cell Lymphoma. N Engl J Med. 2020;382(14):1331–42. 32242358 10.1056/NEJMoa1914347 PMC7731441

[6] Cheah CY, Chihara D, Romaguera JE, Fowler NH, Seymour JF, Hagemeister FB, et al. Patients with mantle cell lymphoma failing ibrutinib are unlikely to respond to salvage chemotherapy and have poor outcomes. Ann Oncol. 2015;26(6):1175–9. 25712454 10.1093/annonc/mdv111

[7] Jain P, Nastoupil L, Westin J, Lee HJ, Navsaria L, Steiner RE, et al. Outcomes and management of patients with mantle cell lymphoma after progression on brexucabtagene autoleucel therapy. Br J Haematol. 2021;192(2):e38–42. 33152104 10.1111/bjh.17197

[8] Pula B, Golos A, Gorniak P, Jamroziak K. Overcoming ibrutinib resistance in chronic lymphocytic leukemia. Cancers (Basel). 2019;11(12):1834. 31766355 10.3390/cancers11121834 PMC6966427

[9] Zhang L, Guo H, Zhang H, Yao YX, Liu Y, Zhang SJ, et al. Genetically defined metabolic targets overcome ibrutinib resistance in mantle cell lymphoma. Blood. 2019;134(Suppl 1):395.31015188

[10] Zhao X, Lwin T, Silva A, Shah B, Tao J, Fang B, et al. Unification of de novo and acquired ibrutinib resistance in mantle cell lymphoma. Nat Commun. 2017;8:14920. 28416797 10.1038/ncomms14920 PMC5399304

[11] Zhang L, Yao Y, Zhang S, Liu Y, Guo H, Ahmed M, et al. Metabolic reprogramming toward oxidative phosphorylation identifies a therapeutic target for mantle cell lymphoma. Sci Transl Med. 2019;11:491. 10.1126/scitranslmed.aau1167 31068440

[12] Zhang S, Jiang VC, Han G, Hao D, Lian J, Liu Y, et al. Longitudinal single-cell profiling reveals molecular heterogeneity and tumor-immune evolution in refractory mantle cell lymphoma. Nat Commun. 2021;12(1):2877. 34001881 10.1038/s41467-021-22872-z PMC8128874

[13] Zheng GX, Terry JM, Belgrader P, Ryvkin P, Bent ZW, Wilson R, et al. Massively parallel digital transcriptional profiling of single cells. Nat Commun. 2017;8:14049. 28091601 10.1038/ncomms14049 PMC5241818

[14] Hao Y, Hao S, Andersen-Nissen E, Mauck Iii WM, Zheng S, Butler A, et al. Integrated analysis of multimodal single-cell data. Cell. 2021;184(13):3573–87. 34062119 10.1016/j.cell.2021.04.048 PMC8238499

[15] Stuart T, Butler A, Hoffman P, Hafemeister C, Papalexi E, Mauck WM, et al. Comprehensive integration of single-cell data. Cell. 2019;177(7):1888–902. 31178118 10.1016/j.cell.2019.05.031 PMC6687398

[16] Tickle TI, Georgescu C, Brown M, Haas B. inferCNV of the Trinity CTAT Project. https://github.com/broadinstitute/inferCNV2019.

[17] Benjamini Y, Hochberg Y. Controlling the false discovery rate: a practical and powerful approach to multiple testing. J R Stat Soc. 1995;57(1):289–300.

[18] Liao Y, Wang J, Jaehnig EJ, Shi Z, Zhang B. WebGestalt 2019: gene set analysis toolkit with revamped UIs and APIs. Nucleic Acids Res. 2019;47(W1):W199–205. 31114916 10.1093/nar/gkz401 PMC6602449

[19] Love MI, Huber W, Anders S. Moderated estimation of fold change and dispersion for RNA-seq data with DESeq2. Genome Biol. 2014;15(12):550. 25516281 10.1186/s13059-014-0550-8 PMC4302049

[20] Cao J, Spielmann M, Qiu X, Huang X, Ibrahim DM, Hill AJ, et al. The single-cell transcriptional landscape of mammalian organogenesis. Nature. 2019;566(7745):496–502. 30787437 10.1038/s41586-019-0969-x PMC6434952

[21] van den Berge K, de Bézieux H, Street K, Saelens W, Cannoodt R, Saeys Y, et al. Trajectory-based differential expression analysis for single-cell sequencing data. Nat Commun. 2020;11(1):1201. 32139671 10.1038/s41467-020-14766-3 PMC7058077

[22] Mounir M, Lucchetta M, Silva TC, Olsen C, Bontempi G, Chen X, et al. New functionalities in the TCGAbiolinks package for the study and integration of cancer data from GDC and GTEx. PLoS Comput Biol. 2019;15(3): e1006701. 30835723 10.1371/journal.pcbi.1006701 PMC6420023

[23] Zhang Y, Park C, Bennett C, Thornton M, Kim D. Rapid and accurate alignment of nucleotide conversion sequencing reads with HISAT-3N. Genome Res. 2021;31:1290–5. 34103331 10.1101/gr.275193.120 PMC8256862

[24] Shumate A, Wong B, Pertea G, Pertea M. Improved transcriptome assembly using a hybrid of long and short reads with StringTie. Plos Comput Biol. 2022;18(6): e1009730. 35648784 10.1371/journal.pcbi.1009730 PMC9191730

[25] Jiang VC, Hao D, Jain P, Li Y, Cai Q, Yao Y, et al. TIGIT is the central player in T-cell suppression associated with CAR T-cell relapse in mantle cell lymphoma. Mol Cancer. 2022;21(1):185. 36163179 10.1186/s12943-022-01655-0 PMC9513944

[26] Jain P, Tang GL, Yin CC, Ok CY, Navsaria L, Badillo M, et al. Complex karyotype is a significant predictor for worst outcomes in patients with mantle cell lymphoma (MCL) treated with BTK Inhibitors - comprehensive analysis of 396 patients. Blood. 2020;136(Suppl 1):32–3.

[27] Liberzon A, Birger C, Thorvaldsdottir H, Ghandi M, Mesirov JP, Tamayo P. The Molecular Signatures Database (MSigDB) hallmark gene set collection. Cell Syst. 2015;1(6):417–25. 26771021 10.1016/j.cels.2015.12.004 PMC4707969

[28] Bacon CW, D’Orso I. CDK9: a signaling hub for transcriptional control. Transcription. 2019;10(2):57–75. 30227759 10.1080/21541264.2018.1523668 PMC6602564

[29] Haghverdi L, Buttner M, Wolf FA, Buettner F, Theis FJ. Diffusion pseudotime robustly reconstructs lineage branching. Nat Methods. 2016;13(10):845–8. 27571553 10.1038/nmeth.3971

[30] Geuens T, Bouhy D, Timmerman V. The hnRNP family: insights into their role in health and disease. Hum Genet. 2016;135(8):851–67. 27215579 10.1007/s00439-016-1683-5 PMC4947485

[31] Paul I, Ahmed SF, Bhowmik A, Deb S, Ghosh MK. The ubiquitin ligase CHIP regulates c-Myc stability and transcriptional activity. Oncogene. 2013;32(10):1284–95. 22543587 10.1038/onc.2012.144

[32] Lee J, Zhang LL, Wu W, Guo H, Li Y, Sukhanova M, et al. Activation of MYC, a bona fide client of HSP90, contributes to intrinsic ibrutinib resistance in mantle cell lymphoma. Blood Adv. 2018;2(16):2039–51. 30115641 10.1182/bloodadvances.2018016048 PMC6113611

[33] Huang A, Garraway LA, Ashworth A, Weber B. Synthetic lethality as an engine for cancer drug target discovery. Nat Rev Drug Discov. 2020;19(1):23–38. 31712683 10.1038/s41573-019-0046-z

[34] Miao W, Li L, Zhao Y, Dai X, Chen X, Wang Y. HSP90 inhibitors stimulate DNAJB4 protein expression through a mechanism involving N(6)-methyladenosine. Nat Commun. 2019;10(1):3613. 31399576 10.1038/s41467-019-11552-8 PMC6688989

[35] Dhanasekaran R, Deutzmann A, Mahauad-Fernandez WD, Hansen AS, Gouw AM, Felsher DW. The MYC oncogene - the grand orchestrator of cancer growth and immune evasion. Nat Rev Clin Oncol. 2022;19(1):23–36. 34508258 10.1038/s41571-021-00549-2 PMC9083341

[36] Wang L, Tang G, Medeiros LJ, Xu J, Huang W, Yin CC, et al. MYC rearrangement but not extra MYC copies is an independent prognostic factor in patients with mantle cell lymphoma. Haematologica. 2021;106(5):1381–9. 32273477 10.3324/haematol.2019.243071 PMC8094099

[37] Ott G, Rosenwald A, Campo E. Understanding MYC-driven aggressive B-cell lymphomas: pathogenesis and classification. Blood. 2013;122(24):3884–91. 24009228 10.1182/blood-2013-05-498329

[38] Dani C, Blanchard JM, Piechaczyk M, El Sabouty S, Marty L, Jeanteur P. Extreme instability of myc mRNA in normal and transformed human cells. Proc Natl Acad Sci USA. 1984;81(22):7046–50. 6594679 10.1073/pnas.81.22.7046 PMC392073

[39] Gregory MA, Hann SR. c-Myc proteolysis by the ubiquitin-proteasome pathway: stabilization of c-Myc in Burkitt’s lymphoma cells. Mol Cell Biol. 2000;20(7):2423–35. 10713166 10.1128/mcb.20.7.2423-2435.2000 PMC85426

[40] Chen H, Liu HD, Qing GL. Targeting oncogenic Myc as a strategy for cancer treatment. Signal Transduct Target Ther. 2018;3:5. 29527331 10.1038/s41392-018-0008-7 PMC5837124

[41] Wang M, Zhao XH, Jiang HJ, Yan JC, Sotomayor E, Shain KH, et al. CDK9 as a new therapeutic vulnerability for ibrutinib resistance in mantle cell lymphoma (MCL). Blood. 2020;136(Suppl 1):34–5.

[42] Poole CJ, Zheng W, Lee H, Young D, Lodh A, Chadli A, et al. Targeting the MYC oncogene in Burkitt lymphoma through HSP90 inhibition. Cancers (Basel). 2018;10(11):448. 30453475 10.3390/cancers10110448 PMC6266960

[43] Jacobson C, Kopp N, Layer JV, Redd RA, Tschuri S, Haebe S, et al. HSP90 inhibition overcomes ibrutinib resistance in mantle cell lymphoma. Blood. 2016;128(21):2517–26. 27742706 10.1182/blood-2016-04-711176

[44] Miao W, Li L, Zhao Y, Dai X, Chen X, Wang Y. HSP90 inhibitors stimulate DNAJB4 protein expression through a mechanism involving N6-methyladenosine. Nat Commun. 2019;10(1):3613. 31399576 10.1038/s41467-019-11552-8 PMC6688989

