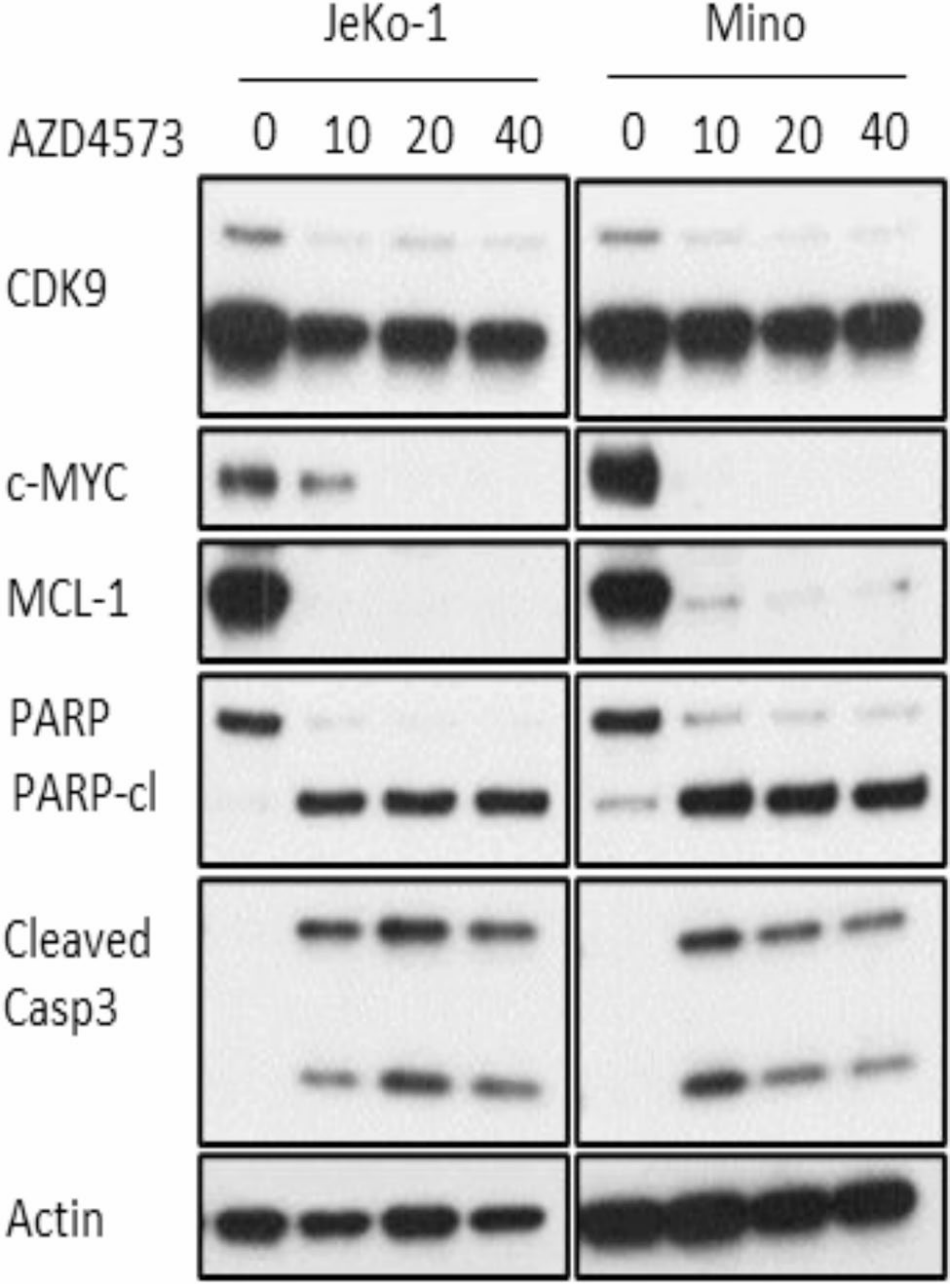# Correction: The HSP90-MYC-CDK9 network drives therapeutic resistance in mantle cell lymphoma

**DOI:** 10.1186/s40164-025-00735-3

**Published:** 2026-02-11

**Authors:** Fangfang Yan, Vivian Jiang, Alexa Jordan, Yuxuan Che, Yang Liu, Qingsong Cai, Yu Xue, Yijing Li, Joseph McIntosh, Zhihong Chen, Jovanny Vargas, Lei Nie, Yixin Yao, Heng-Huan Lee, Wei Wang, JohnNelson R. Bigcal, Maria Badillo, Jitendra Meena, Christopher Flowers, Jia Zhou, Zhongming Zhao, Lukas M. Simon, Michael Wang

**Affiliations:** 1 Center for Precision Health, School of Biomedical Informatics, The University of Texas Health Science Center at Houston, Houston, TX 77030 USA; 2 Department of Lymphoma and Myeloma, The University of Texas MD Anderson Cancer Center, Houston, TX USA; 3 Department of Pharmacology and Toxicology, University of Texas Medical Branch, Galveston, TX 77555 USA; 4 Department of Biochemistry and Molecular Biology, Verna and Marrs McLean, Baylor College of Medicine, Houston, TX 77030 USA; 5 Human Genetics Center, School of Public Health, The University of Texas Health Science Center at Houston, Houston, TX 77030 USA; 6 MD Anderson Cancer Center UTHealth Graduate School of Biomedical Sciences, Houston, TX 77030 USA; 7 Therapeutic Innovation Center, Baylor College of Medicine, Houston, TX 77030 USA; 8 Department of Stem Cell Transplantation and Cellular Therapy, The University of Texas MD Anderson Cancer Center, Houston, TX USA

**Correction: Experimental Hematology & Oncology (2024) 13:14** 10.1186/s40164-024-00484-9

After online publication of the article [1], the authors notified that Figure S9C in the published version does not appear as intended. Unfortunately, this discrepancy was not identified during the production process.

Specifically, the current version of Figure S9C displays identical western data for both JeKo and Mino cell lines, which is incorrect. Published version and correct version of the supplementary Figure S9c is given below:

Published version


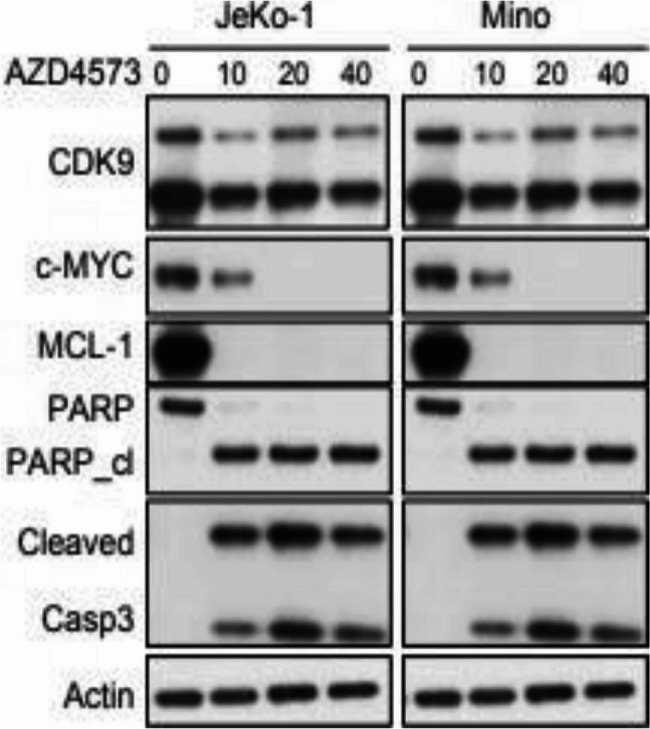


Correct version