# Supplementary material for: The HSP90-MYC-CDK9 network drives therapeutic resistance in mantle cell lymphoma
Source: Exp Hematol Oncol. 2024 Feb 7;13:14. doi: 10.1186/s40164-024-00484-9 (PMC10848414; doi:10.1186/s40164-024-00484-9)
Supplement: Supplementary file 5 — Additional file 5. Additional figures. Figure S1. Data integration removes batch effects. Figure S2. Tumor B cells exhibit increased inter-patient heterogeneity compared to the tumor microenvironment. Figure S3. Tumor B cells carry more CNV aberrations compared to non-tumor T cells. Figure S4. Higher genome instability scores associate with sequential resistance to BTKi and CAR-T therapy. Figure S5. Mixed model detected outcome-specific gene signatures across multiple patients and samples. Figure S6. Histograms showing distribution of p-values derived from our mixed regression models. Figure S7. Outcome-specific gene signature was validated in an independent bulk RNA-seq MCL dataset. Figure S8. Positive Correlation between MYC and HSP90AB1 is tissue-specific and only in lymphoma cell lines. Figure S9. Altered pathways upon single treatment of CDK9 or HSP90 inhibitors. Figure S10. Enriched pathways upon single inhibitor treatment. Figure S11. The combination effect of CDK9 or HSP90 inhibitors were validated in five additional MCL cell lines. [file 40164_2024_484_MOESM5_ESM.pdf]

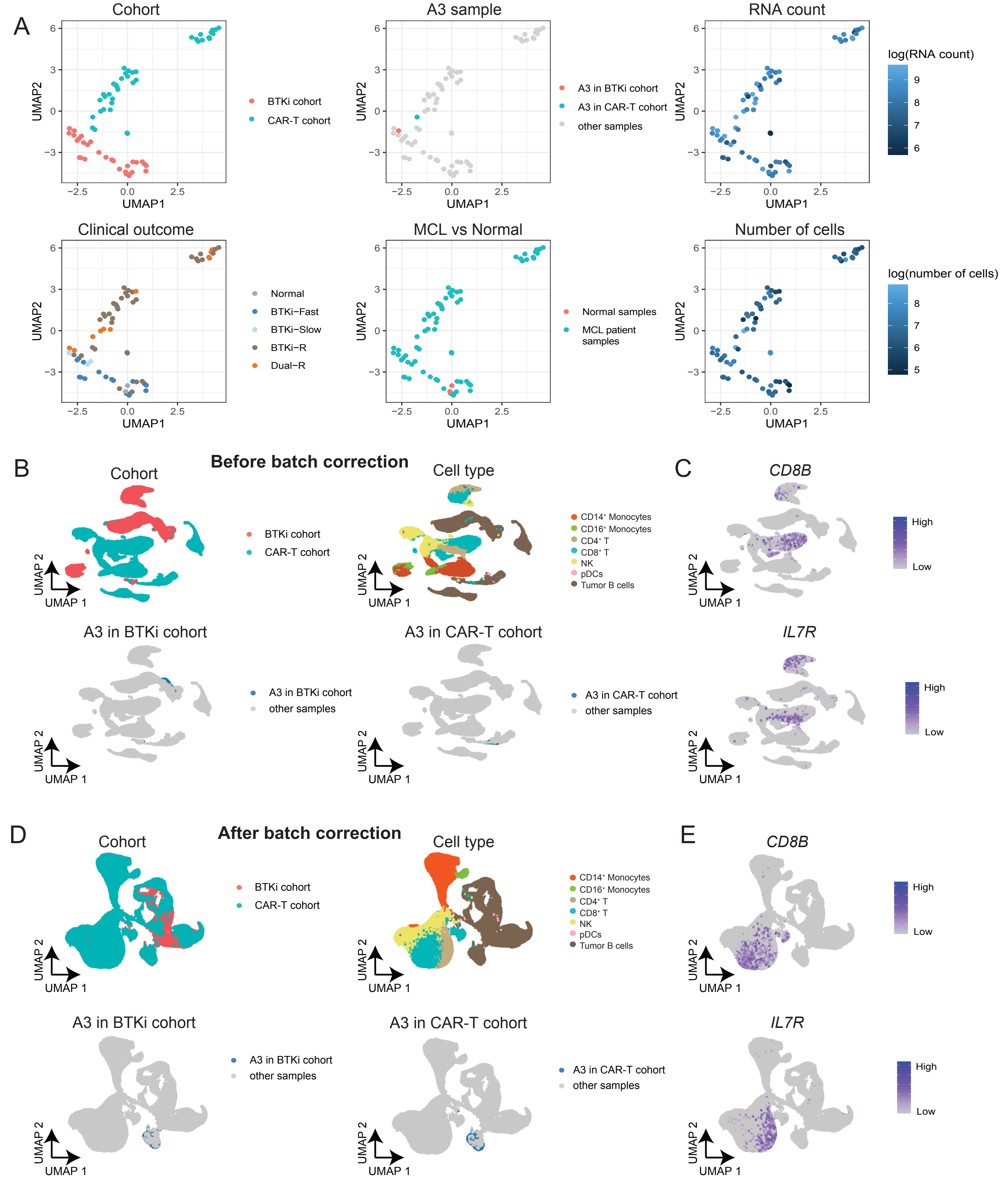

Supplemental Figure S1. Data integration removes batch effects. (A) UMAP dimension reduction of *in silico* bulk samples reveals that the cohort variable is driving a large proportion of variance and thus batch correction is necessary. **(B)** UMAP visualization of single-cell RNA-seq data before batch correction colored by cohort and inferred cell type. Sample A3 was profiled in both cohorts and maps into distinct regions of the embedding. **(C)** Feature plot of select marker genes before batch correction. **(D)** UMAP visualization of single-cell RNA-seq data after batch correction colored by cohort and inferred cell type. Sample A3 was profiled in both cohorts and cells overlap. **(E)** Feature plot of marker genes after batch correction.

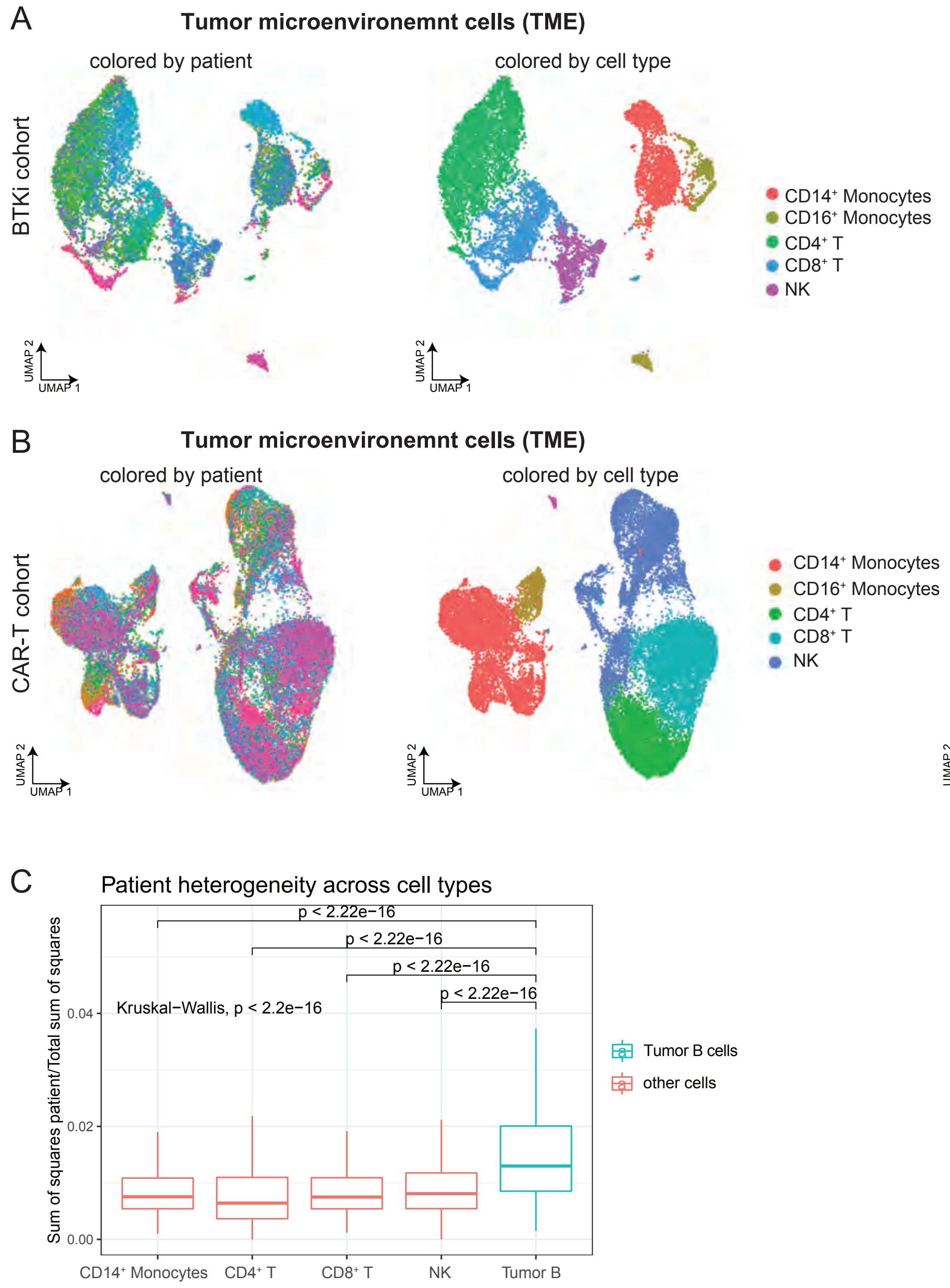

Supplemental Figure S2. Tumor B cells exhibit increased inter-patient heterogeneity compared to the tumor microenvironment. UMAP visualizations of tumor B cells (left, colored by patient), TME cells (middle, colored by patient) and TME cells (right, colored by cell types) from the BTKi cohort **(A)** and CAR-T cohort **(B).** Tumor B cells cluster by patient while TME cells cluster by cell type. **(C)** Boxplot shows the proportion of variance explained by the patient variable (y-axis) across cell types (x-axis). Tumor B cells show statistically significant increase of patient heterogeneity (blue) compared to TME cell types (red).

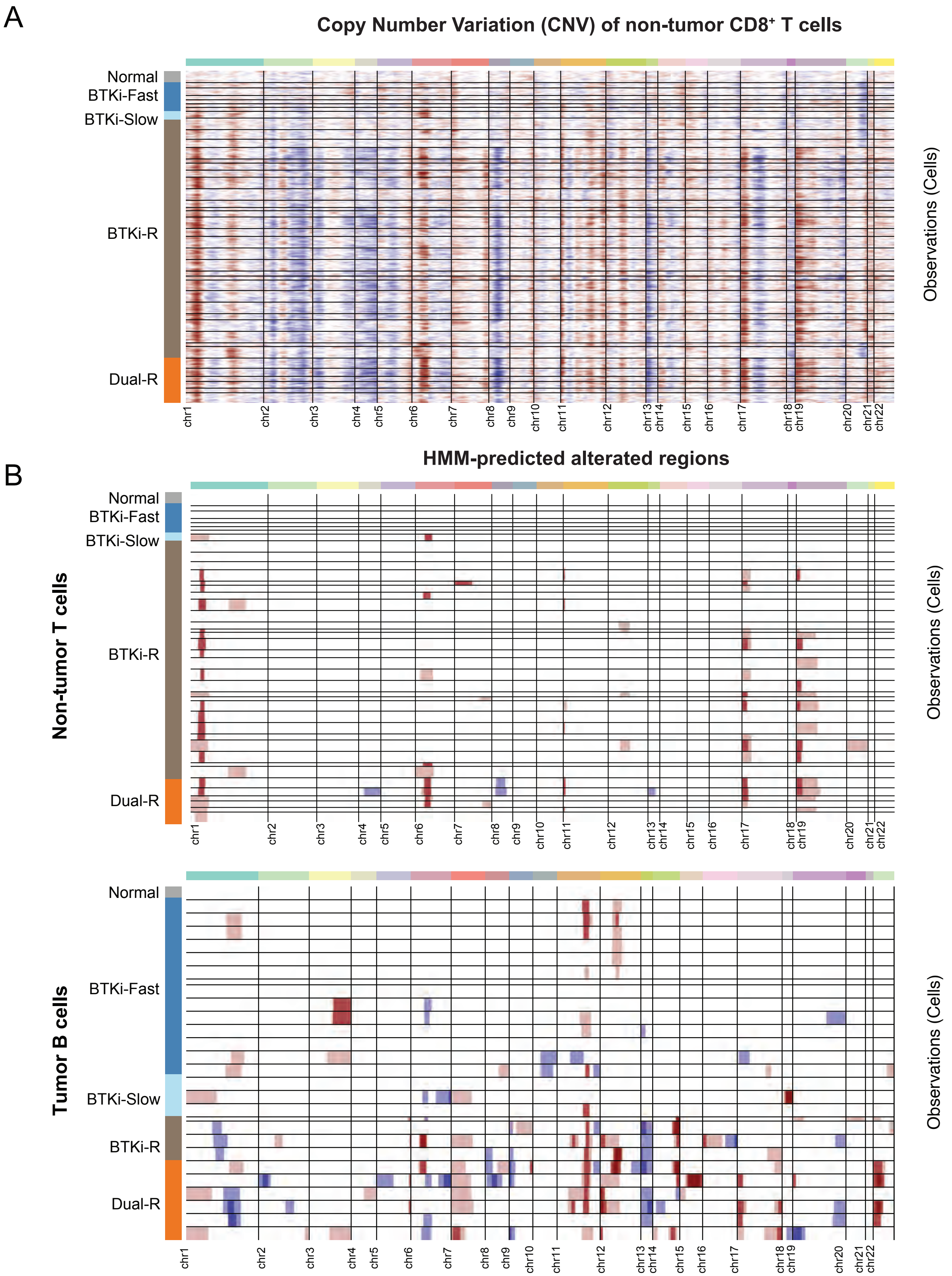

Supplemental Figure S3, Tumor B cells carry more CNV aberrations compared to non-tumor T cells. (A) Heatmap shows the CNVs in each sample (rows) across chromosomes (columns) in non-tumor $CD8^+$ T cells. Color represents gains (red) or losses (blue). Bars on the top of the heatmap indicate chromosomes from 1 to 22 and relative length. Bars on the left indicate sample names and clinical outcomes. Samples were ordered by tumor aggressiveness from top (Normal) to bottom (Dual-R). Compared to tumor B cells (Figure 2B), much less CNVs were observed. **(B)** Heatmap shows the HMM predicted altered regions (BayesMaxPNormal = 0.2) for non-tumor T cells (upper panel) and tumor B cells (bottom panel), respectively. Many more altered regions were identified in B cells compared to T cells.

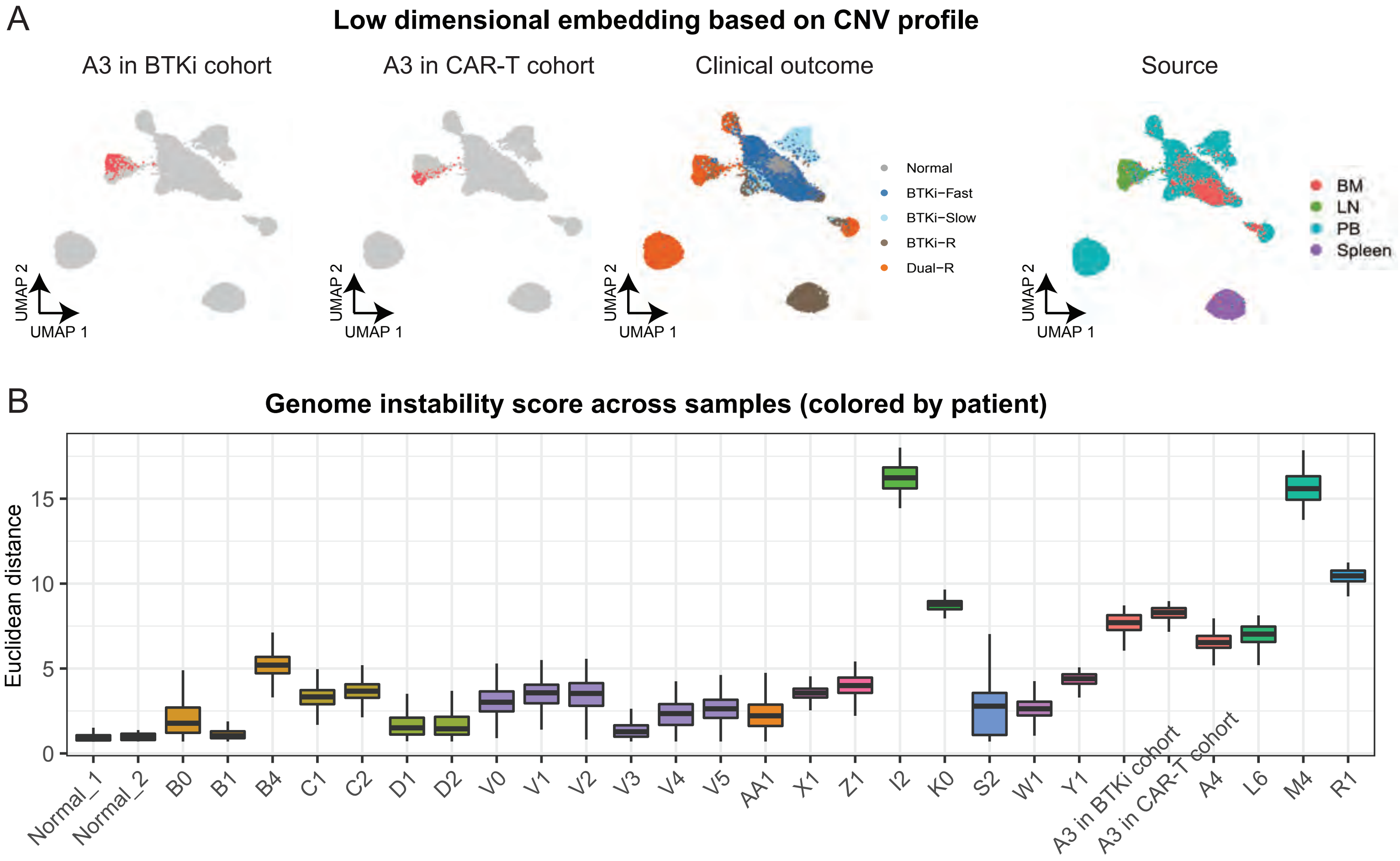

Supplemental Figure S4, Higher genome instability scores associate with sequential resistance to BTKi and CAR-T therapy. (A) UMAP embedding plot based on CNV profiles colored by patient A3 in cohort1 and cohort2, clinical outcome, and source, respectively. The A3 sample, profiled in both cohorts, showed similar genomic instability profiles demonstrating the robustness of this metric across cohorts. Normal samples were surrounded by BTKi-Fast and BTKi-Slow samples in the embedding space, followed by BTKi-R and Dual-R samples. **(B)** Boxplot shows the genome instability score (y-axis) across samples (x-axis). Dual samples had the highest score. Most patients had higher genome instability scores across time, except patient V.

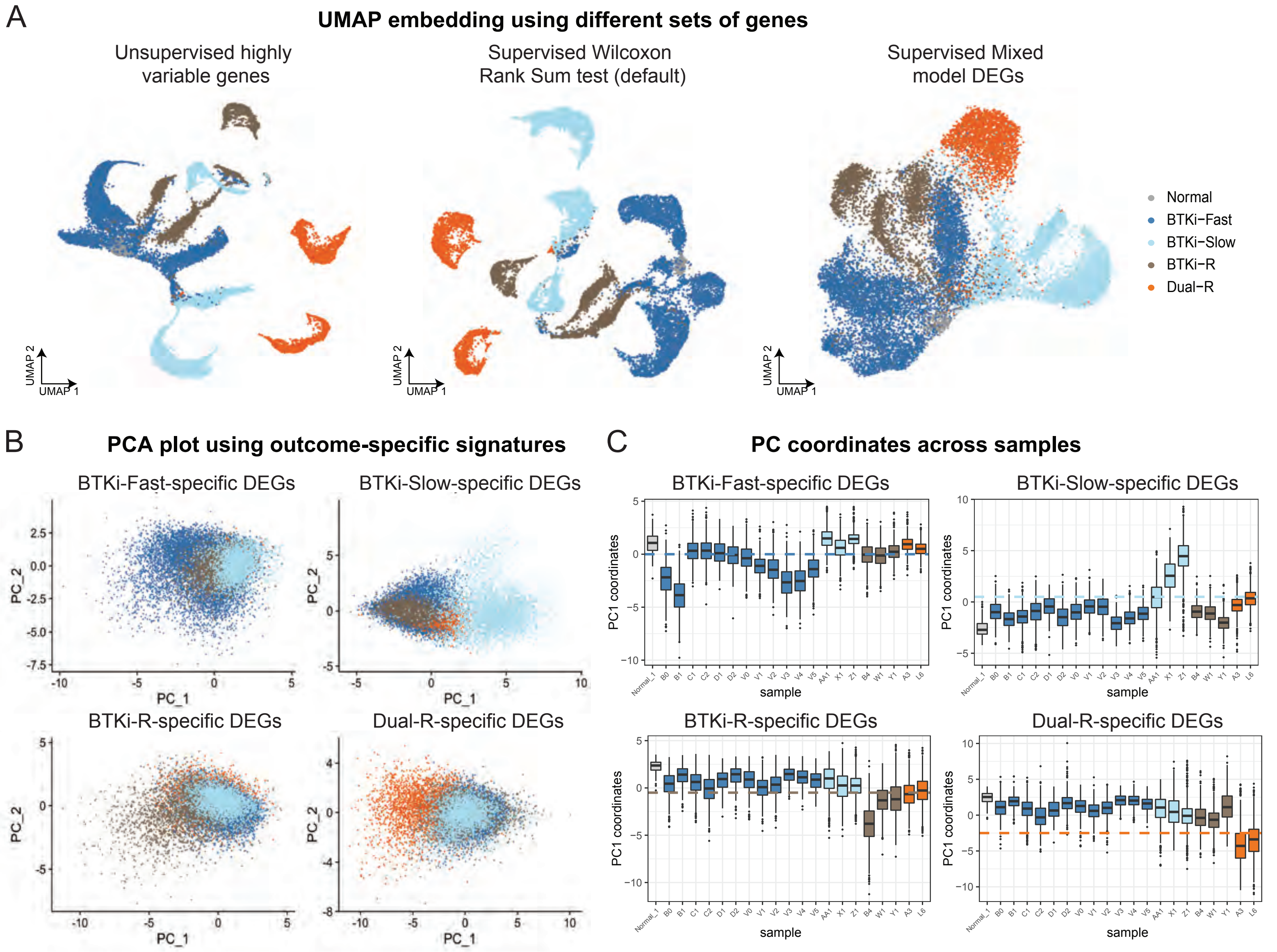

Supplemental Figure S5, Mixed model detected outcome-specific gene signatures across multiple patients and samples. (A) UMAP embedding using different sets of genes. Left: highly variable genes; Middle: differentially expressed genes between clinical outcomes using default Wilcoxon Rank Sum test; Right: differentially expressed genes between clinical outcomes using the mixed model. Color represents clinical outcomes. **(B)** Scatter plot shows PC1 (x-axis) and PC2 (y-axis) using outcome-specific signatures. **(C)** Boxplot shows the first PC coordinate (y-axis) across different samples (x-axis).

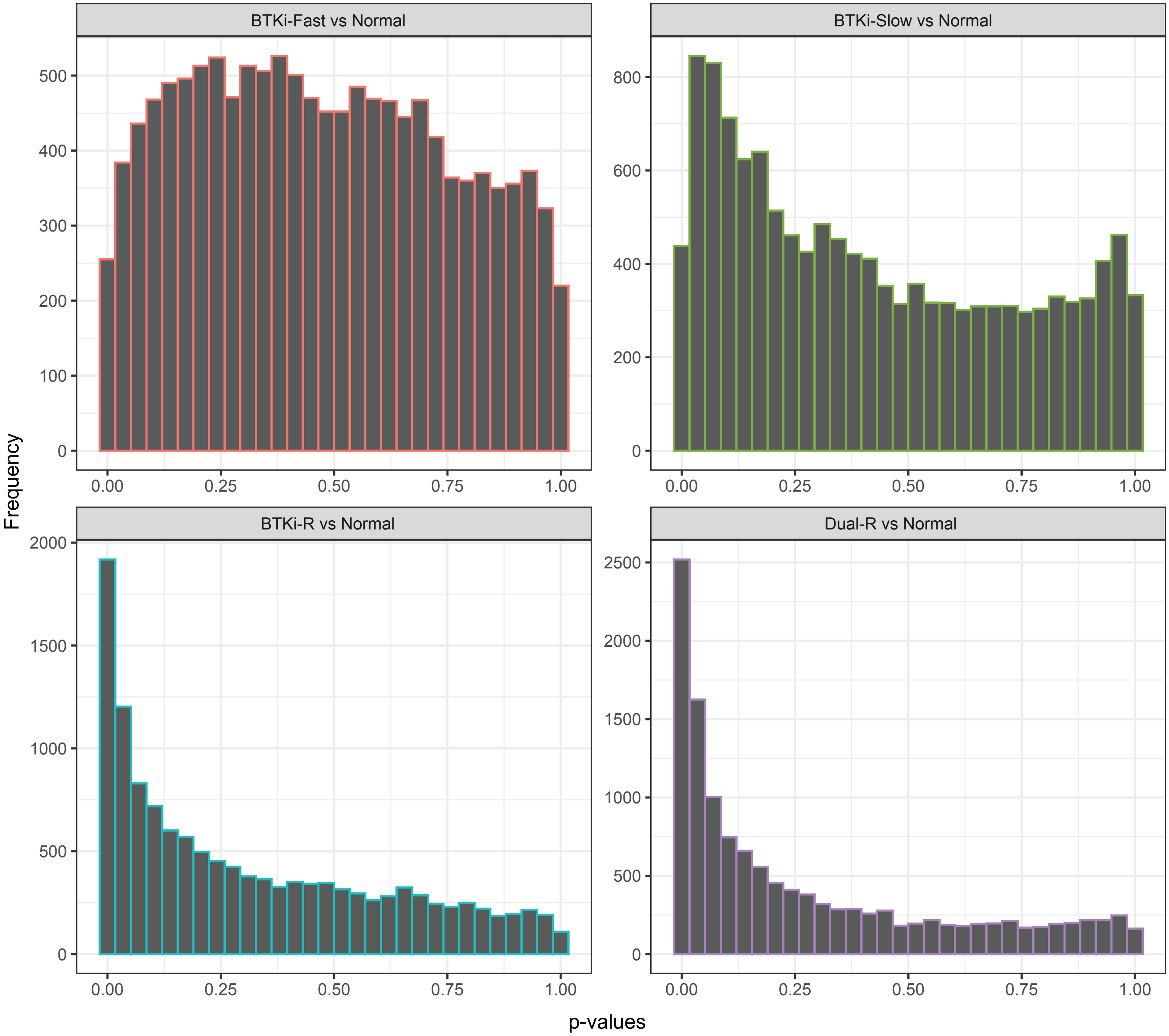

Supplemental Figure S6, Histograms showing distribution of p-values derived from our mixed regression models. We observed strong enrichment of low p-values, indicating the presence of statistical signal that goes beyond spurious associations.

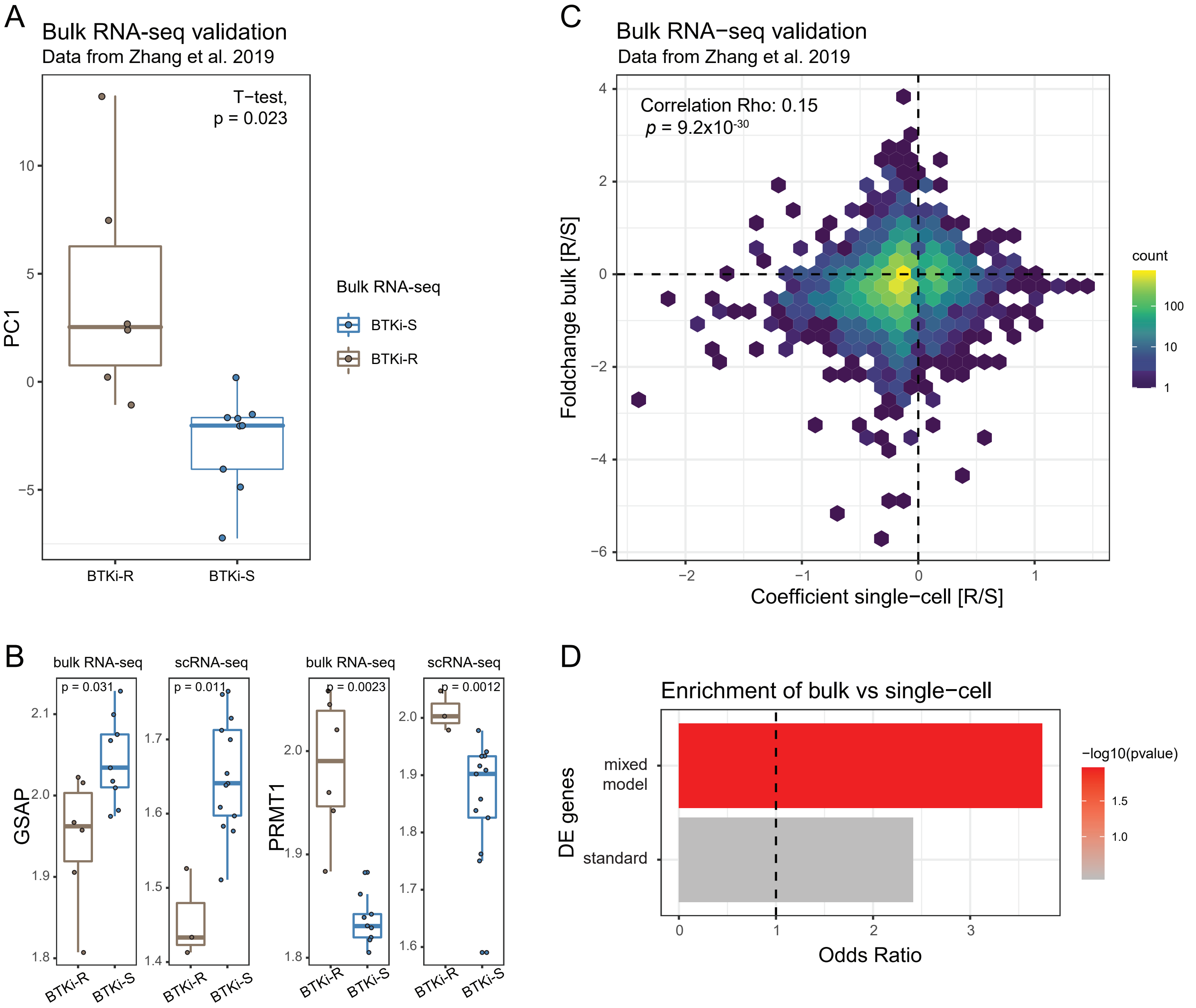

Supplemental Figure S7, Outcome-specific gene signature was validated in an independent bulk RNA-seq MCL dataset. (A) Boxplot shows principle component one separating ibrutinib-resistant and sensitive samples from Zhang et al bulk RNA-seq. Color represents ibrutinib sensitivity. **(B)** Boxplot shows the expression of two representative differentially expressed genes, GSTP1 and PRMT1, at the bulk (left) and single-cell levels (right). **(C)** Scatter plot shows significant correlation of the model coefficient from scRNA-seq (x-axis) and fold changes derived from bulk RNA-seq (y-axis). A positive coefficient represents greater expression in IBR resistant compared to sensitive in our scRNA-seq data. A positive fold change represents greater expression in IBR resistant compared to IBR sensitive samples in the published bulk RNA-seq data. **(D)** Bar graph shows enrichment odds ratio of single-cell derived differentially expressed genes compared to differentially expressed genes derived from the bulk data. Compared to the standard Wilcoxon test, the mixed model identified genes with a significant overlap in the bulk data. Top bar (red): mixed model derived scRNA-seq. Bottom bar (grey): standard model derived scRNA-seq differentially expressed genes.

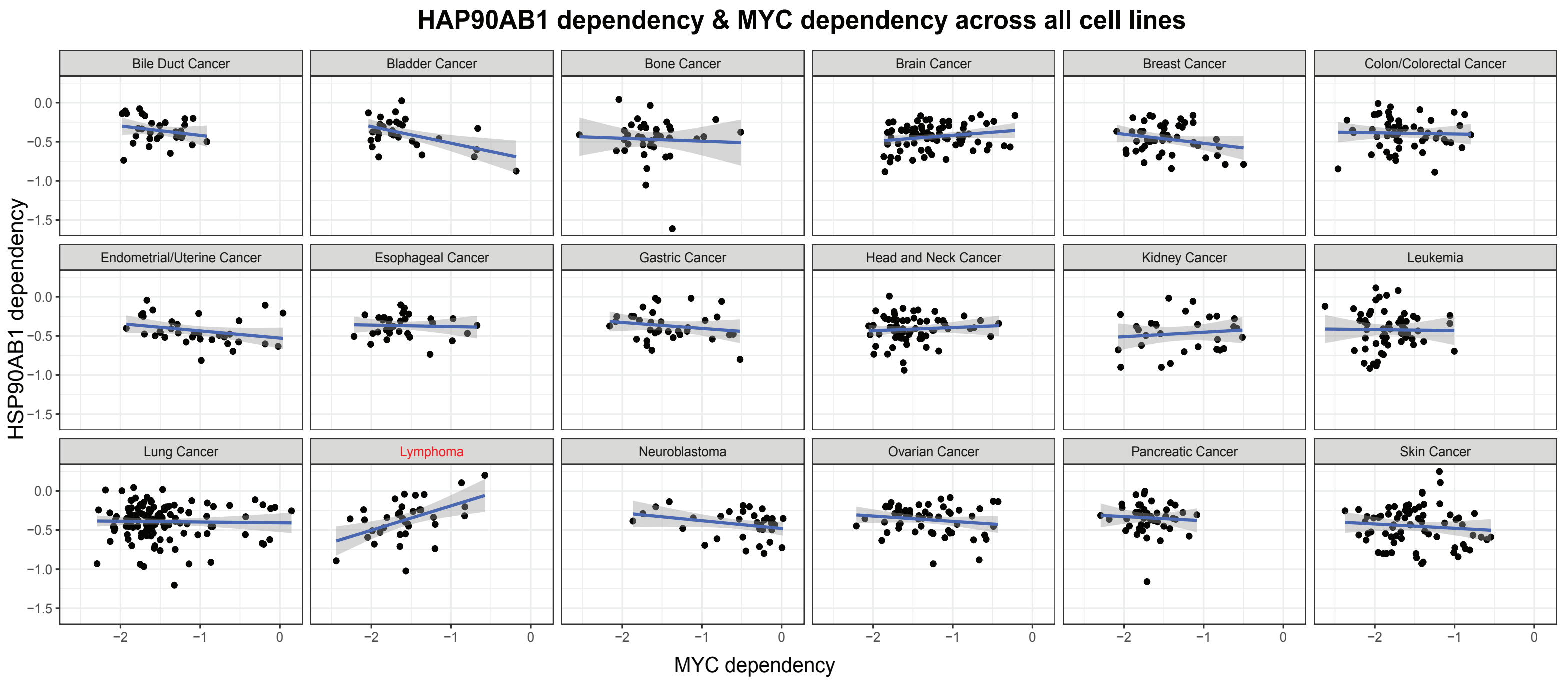

Supplemental Figure S8. Positive Correlation between MYC and HSP90AB1 is tissue-specific and only in lymphoma cell lines. Scatter plot with fitted smooth lines show the correlation between HSP90AB1 dependency (y-axis) and MYC dependency (x-axis) across different disease cell lines. The significant correlation between MYC and HSP90AB1 dependencies exclusively show up in lymphoma cell lines.

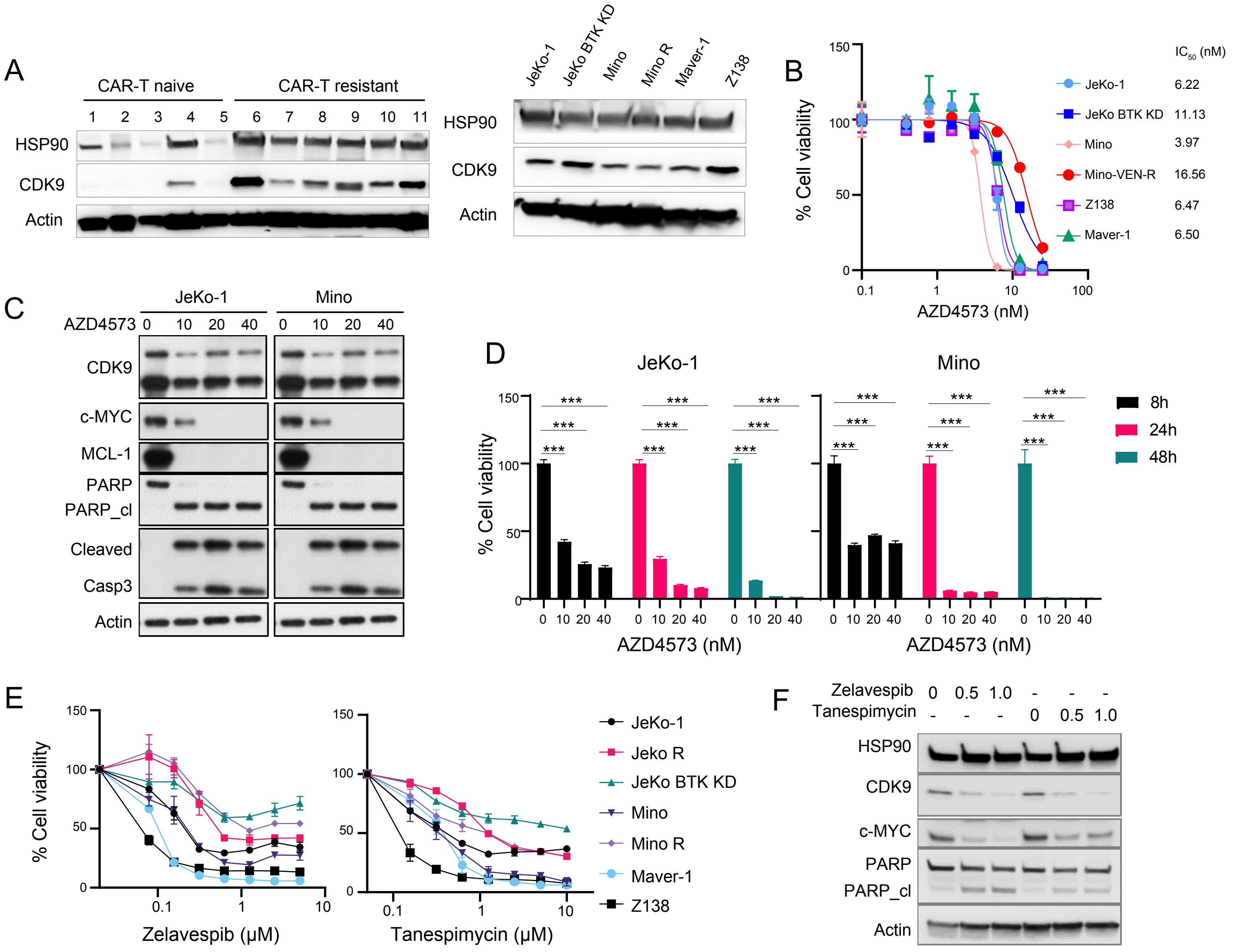

Supplemental Figure S9. Altered pathways upon single treatment of CDK9 or HSP90 inhibitors. (A) Western blot shows HSP90 and CDK9 protein expression in CAR-T naïve and resistant primary patient samples (left) and MCL cell lines (right). **(B)** CDK9 inhibition potently suppressed the cell viability in all 6 MCL cell lines upon treatment with AZD4573 for 72 h. **(C)** Proteins altered in JeKo-1 cells and Mino cells after treatment with AZD4573 at 0, 10, 20, and 40 nM for 8 h. **(D)** Cell viability was suppressed in JeKo-1 and Mino cells after treatment with AZD4573 at 0, 10, 20, and 40 nM for 8 h, 24 h, and 48 h, respectively. **(E)** HSP90 inhibitors zelavespib or tanespimycin inhibited cell viability. **(F)** Zelavespib or tanespimycin suppressed expression of CDK9 and MYC and induced cleavage of PARP in Z138 cells upon treatment for 24 h.

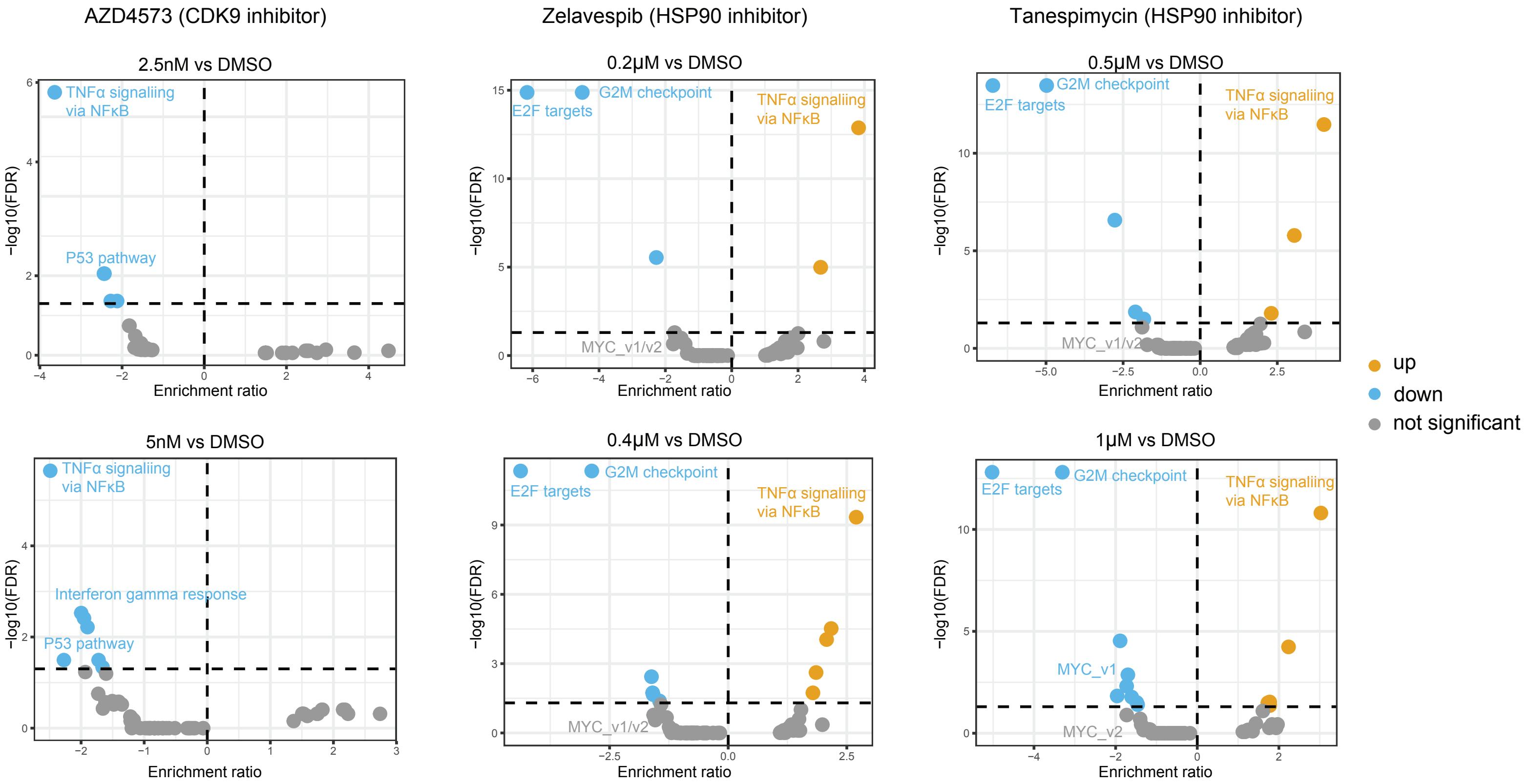

Supplemental Figure S10. Enriched pathways upon single inhibitor treatment. Volcano plots show the log Fold change (x-axis) and -log10 (FDR) (y-axis) of enriched pathways upon AZD4573 (left), zelavespib (middle), and tanespimycin (right) treatments, respectively. Each dot represents an enriched pathway and is colored by significance (up: yellow, down: blue, not significant: grey).

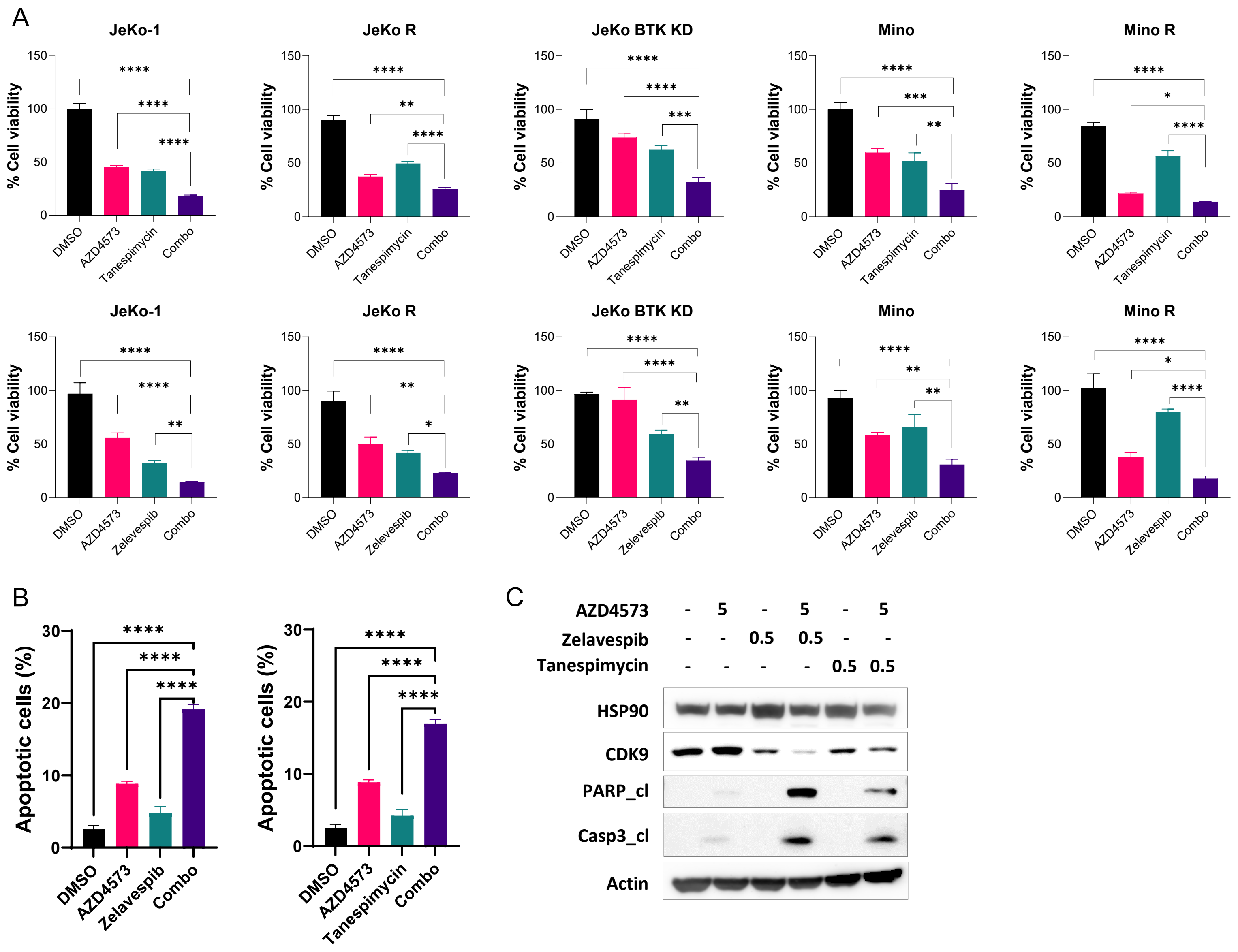

Supplemental Figure S11. The combination effect of CDK9 or HSP90 inhibitors were validated in five additional MCL cell lines. (**A**) AZD4573 in combination with tanespimycin (top panels) or zelavespib (bottom panels) synergistically suppressed cell viability in JeKo-1, JeKo-R, JeKo BTK KD, Mino, and Mino-R cells upon treatment for 24 h. (**B**) AZD4573 in combination with zelavespib (left panel) or tanespimycin (right panel) synergistically apoptosis in Mino cells upon treatment for 24h. (**C**) AZD4573 in combination with zelavespib or tanespimycin dramatically enhanced the induction of apoptosis by either single agent in Mino cells upon treatment for 24h. *, $P < 0.05$; **, $P < 0.01$; ***, $P < 0.001$; ****, $P < 0.0001$.
